# Development and validation of an m6A and autophagy related lncRNAs signature for predicting survival and modulating the immune microenvironment in esophageal squamous cell carcinoma

**DOI:** 10.3389/fimmu.2026.1766278

**Published:** 2026-05-28

**Authors:** Mingyi Yang, Honghao Ren, Jing Hu, Pengfei Wen, Jiale Xie, Xianjie Wan, Lin Liu, Zhi Yang, Ming Zhang, Xiaodong Ren, Yani Su

**Affiliations:** 1 Department of Joint Surgery, HongHui Hospital, Xi’an Jiaotong University, Xi’an, Shaanxi, China; 2 Department of Radiotherapy, Tangdu Hospital, The Fourth Military Medical University, Xi’an, Shaanxi, China; 3 Department of General Practice, Honghui Hospital, Xi’an Jiaotong University, Xi’an, Shaanxi, China

**Keywords:** autophagy, esophageal squamous cell carcinoma, immune microenvironment, M6A, prognosis

## Abstract

**Objective:**

This research is designed to establish a prognostic framework derived from m6A- and autophagy-related lncRNAs (m6aARLncs) to enhance survival prediction in esophageal squamous cell carcinoma (ESCC). Concurrently, it endeavors to elucidate the role of prognostic framework in modulating the tumor immune microenvironment.

**Methods:**

Transcriptomic data from The Cancer Genome Atlas (TCGA) and the Gene Expression Omnibus (GEO) were employed as the training and independent validation cohorts, respectively. m6A-related genes (m6aRGs) and autophagy-related genes (ARGs) were curated from published literature and the Human Autophagy Database (HADb), respectively. Subsequently, differentially expressed m6aARLncs (DE-m6aARLncs) were identified by integrating co-expression analysis with differential expression profiling. A prognostic risk signature was constructed using univariate Cox and LASSO regression analyses. The model’s predictive efficacy was rigorously validated through risk heatmap, survival analysis, ROC curves, differential analysis and independent prognostic analysis. Furthermore, the association between riskScores and clinical features was assessed. To elucidate the model’s biological relevance, Gene Set Enrichment Analysis (GSEA) was performed. The impact of the risk signature on the tumor immune microenvironment was comprehensively evaluated via tumor microenvironment analysis, immune cell correlation analysis, single-sample gene set enrichment analysis (ssGSEA), and immune checkpoint profiling. Single-cell sequencing data analysis was carried out. Drug sensitivity profiling was conducted to identify putative therapeutic agents tailored to distinct risk subgroups. The putative m6A-autophagy-lncRNA regulatory axis may implicated in ESCC pathogenesis was proposed. Finally, the mRNA expression levels of m6aARLncs were validated using reverse transcription quantitative polymerase chain reaction (RT-qPCR).

**Results:**

We established a robust risk prognostic model based on five m6aARLncs (LINC00847, UBL7-AS1, LINC01554, LINC00601, and FAM222A-AS1), which demonstrated significant efficacy in predicting overall survival in ESCC patients. Comprehensive immune profiling delineated the unique immune phenotype of the prognostic model, characterized by the infiltration of mast cells, B cells, neutrophils, plasmacytoid dendritic cells (pDCs), helper T cells, and tumor-infiltrating lymphocytes (TILs), as well as human leukocyte antigens (HLA), etc. The B cells, neutrophils and dendritic cells identified by immune microenvironment analysis results may have been verified to some extent through single-cell sequencing analysis. Furthermore, the expression of two immune checkpoint molecules, TNFRSF18 and LAIR1, was significantly correlated with risk stratification, suggesting their potential therapeutic relevance. Pharmacogenomic analysis identified nine compounds (Shikonin, Bicalutamide, Bryostatin-1, Epothilone-B, JNK-9L, LFM-A13, QS11, VX-680, and Z-LLNle-CHO) exhibiting differential sensitivity between the dichotomous risk strata. We propose novel m6A-autophagy-lncRNA regulatory axis implicating the 5 m6aARLncs, 5 m6aRGs and 21 ARGs, which may play a pivotal role in ESCC pathogenesis. Finally, the results of RT-qPCR verification indicated that the expressions of FAM222A-AS1, LINC00601, LINC00847, LINC01554 and UBL7-AS1 were upregulated.

**Conclusion:**

This study yields findings that hold significant clinical implications for refining prognostic stratification and deciphering the immune landscape in ESCC. Moreover, they offer novel perspectives that may pave the way for more precise prognostic assessment and the formulation of targeted therapeutic interventions.

## 1 Introduction

Esophageal cancer (EC) represents one of the most aggressive and lethal malignancies, posing significant challenges in clinical management and patient outcomes. Histologically, EC is primarily categorized into two major subtypes: esophageal squamous cell carcinoma (ESCC) and esophageal adenocarcinoma (EAC), which exhibit distinct etiological, molecular, and pathological characteristics, thereby necessitating divergent therapeutic strategies and yielding disparate prognostic outcomes (1). Among these, ESCC constitutes the predominant histological subtype, accounting for nearly 90% of global EC cases, and ranks as the sixth leading cause of cancer-associated mortality worldwide (2). The aggressive nature of ESCC is further compounded by its frequent late-stage diagnosis (3). The insidious onset and rapid progression of ESCC contribute to its advanced detection, where therapeutic options are largely limited to multimodal approaches, including surgery, chemoradiotherapy, and systemic chemotherapy, albeit with suboptimal efficacy (2). Moreover, the absence of well-defined molecular targets further exacerbates the poor prognosis of ESCC patients, underscoring the critical need for elucidating its underlying pathogenic mechanisms to facilitate the discovery of novel and actionable therapeutic targets (4).

N6-methyladenosine (m6a), the most prevalent and extensively studied RNA modification, plays a pivotal role in post-transcriptional gene regulation by modulating RNA stability, translational efficiency, alternative splicing, and nuclear export processes (5). Accumulating evidence has established a strong association between m6a methylation and oncogenesis, with numerous studies demonstrating its regulatory influence on cancer cell proliferation, invasion, and metastatic potential (6). Beyond its role in transcriptional control, m6a serves as a critical metabolic regulator in tumorigenesis, where m6a methyltransferases, demethylases, and reader proteins orchestrate metabolic reprogramming—a hallmark of cancer progression (7). Notably, dysregulated m6a modification and its associated regulatory machinery have been implicated in the pathogenesis, progression, therapeutic response, and clinical outcomes of EC (8). For instance, the m6a demethylase fat mass and obesity-associated protein has been shown to drive tumorigenesis in EC by reprogramming lipid metabolism, highlighting the mechanistic link between epitranscriptomic modifications and metabolic dysregulation in cancer (9, 10). Given these findings, elucidating the specific roles of m6a regulatory factors in EC could deepen our understanding of its molecular pathogenesis and pave the way for novel diagnostic biomarkers, precision therapeutic strategies, and improved prognostic assessments for EC patients.

Autophagy, a conserved form of type II programmed cell death, serves as a double-edged sword in oncogenesis, exhibiting context-dependent roles that vary across stages of tumor development. During early tumorigenesis, autophagy functions as a critical quality-control mechanism, eliminating damaged organelles and misfolded proteins to maintain cellular homeostasis, thereby acting as a tumor-suppressive barrier against malignant transformation and progression. However, in advanced malignancies, autophagy is co-opted by established tumors as an adaptive survival mechanism under metabolic and therapeutic stress, facilitating nutrient recycling, sustaining proliferation, and fostering metastatic dissemination (11). This biphasic functionality underscores the therapeutic potential of autophagy modulation as a dynamic intervention strategy in cancer treatment (11). In EC, particularly ESCC, autophagy further influences the tumor immune microenvironment (TIME). Notably, radiotherapy-induced autophagy has been shown to enhance immune cell maturation, migration, and tumor infiltration through multifaceted mechanisms. This immunomodulatory shift potentiates the synergistic efficacy of combined radiotherapy and immunotherapy, offering a promising avenue for improving therapeutic outcomes (12). Given these pivotal roles, elucidating the precise mechanisms by autophagy shapes the immune landscape of ESCC holds significant translational implications for developing novel combinatorial treatment regimens.

**Figure 1:**
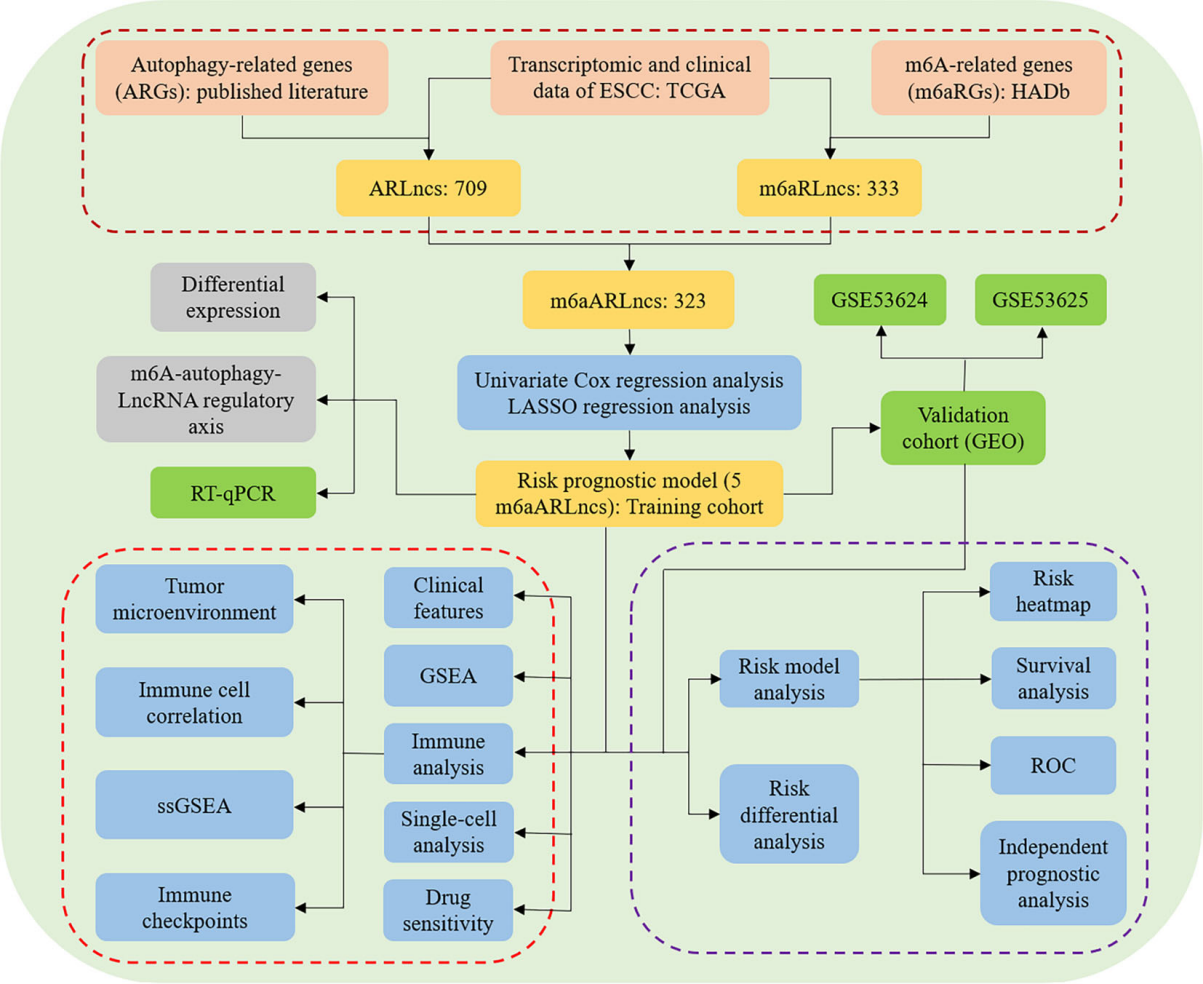
The flowchart of this study.

The development of robust tumor risk prediction models holds substantial clinical value for prognostic assessment and the optimization of precision oncology strategies. Recent years have witnessed significant progress in prognostic modeling for EC, with several novel predictive frameworks demonstrating improved accuracy in outcome prediction (13, 14). However, despite these advancements, the field lacks dedicated risk stratification models that systematically incorporate the emerging biomarkers m6a RNA modification and autophagy processes in ESCC - a critical knowledge gap that warrants urgent investigation. The present study addresses this unmet need by constructing an innovative prognostic model that integrates m6a and autophagy into a unified risk assessment framework for ESCC. Our model not only establishes the combined prognostic value of these molecular pathways but also elucidates their collective impact on tumor immunomodulation. This work provides novel insights into the synergistic roles of m6a modification and autophagy in ESCC pathogenesis, immune microenvironment remodeling, and therapeutic vulnerability. The schematic representation of our research design and analytical pipeline is presented in **Figure 1**, illustrating the systematic approach employed in model construction and validation.

## 2 Materials and methods

### 2.1 Data acquisition and collation

In this investigation, transcriptomic data from The Cancer Genome Atlas (TCGA) (https://portal.gdc.cancer.gov/) served as the primary training cohort, encompassing 81 ESCC specimens alongside 11 normal tissue samples. This dataset provided comprehensive clinicopathological annotations, including gender, age, TNM staging, and overall clinical stage. For independent external validation, the GSE53624 and GSE53625 dataset was procured from the Gene Expression Omnibus (GEO) repository (https://www.ncbi.nlm.nih.gov/geo/). The GSE53624 (Validation 1) comprising 119 tumor-normal paired samples with associated clinical metadata such as age, T stage, N stage, and clinical stage. The GSE53625 (Validation 2) comprising 179 tumor-normal paired samples with associated clinical metadata such as age, T stage, N stage, and clinical stage. The 23 m6A-related genes (m6aRGs) were curated from a previously established publication (15). Furthermore, 222 autophagy-related genes (ARGs) were systematically retrieved from the Human Autophagy Database (HADb) (https://autophagy.lu/v1/).

### 2.2 m6a and autophagy-related LncRNAs related to ESCC

To identify m6A- and autophagy-related LncRNAs (m6aARLncs) relevant to ESCC, we first performed co-expression analysis between 23 m6aRGs and LncRNAs within the ESCC transcriptome using the limma package in R, yielding a set of m6a-related LncRNAs (m6aRLncs). Subsequently, we applied the same analytical approach to examine co-expression patterns between 222 ARGs and LncRNAs, thereby identifying autophagy-related LncRNAs (ARLncs). Both analyses employed stringent statistical criteria, |Pearson correlation coefficient| > 0.3 and P < 0.001, to ensure robust associations. Finally, the intersection of ESCC-related m6aRLncs and ARLncs was taken to derive a set of m6aARLncs implicated in ESCC.

### 2.3 Construction of risk prognostic model

Prognostic m6aARLncs in ESCC were initially identified through univariate Cox regression analysis (P < 0.05) conducted with the survival package in R. Differential expression analysis was subsequently performed using the limma package, accompanied by visualization of expression patterns through heatmaps, leading to the identification of differentially expressed m6aARLncs (DE-m6aARLncs). To enhance model generalizability and reduce overfitting, LASSO regression analysis was implemented via the glmnet package in R, with feature coefficient distributions illustrated using bar charts. The prognostic model underwent independent validation utilizing the GSE53624 and GSE53625 cohorts from the GEO database. Risk stratification models were developed for both training and validation cohorts, where individual riskScores were derived from a linear combination of expression levels of prognostic DE-m6aARLncs. The riskScore was calculated according to the following formula:


$$\text{riskScore}=\sum_{i=1}^{n}(lncrnaexp_{i}\times coef_{i})$$

During riskScore calculation, “n” corresponds to the total number of prognostically significant DE-m6aARLncs identified in ESCC. The index “i” refers to the ith such DE-m6aARLncs significantly associated with ESCC prognosis, while “coef” denotes the regression coefficient derived from the model. For each patient, the riskScore was computed as the linear combination of the expression levels of these DE-m6aARLncs, each weighted by its corresponding regression coefficient. Upon computation, patients were dichotomized into high- and low-risk subgroups based on the median riskScore of the cohort, thus facilitating a binary prognostic classification.

### 2.4 Validation of the risk prognostic model

In the preliminary analytical phase, an integrated risk−stratified heatmap was generated for both the training and validation cohorts using the R software environment. Subsequent survival analyses, implemented through the survival and survminer packages in R, quantitatively assessed differential survival patterns among comparative patient subgroups. Moreover, receiver operating characteristic (ROC) curve analysis was applied to examine the predictive accuracy of the riskScore model in comparison with conventional clinical parameters. A systematic evaluation of the expression profiles of DE-m6aARLncs included in the prognostic risk model was conducted through comparative intergroup analyses between high- and low-risk patients in both training and validation cohorts. Differential expression patterns were graphically represented using box plots generated with the “reshape2” and “ggpubr” packages in R. Univariate and multivariate Cox regression analyses were performed using the survival package in R to evaluate whether the riskScore could serve as an independent prognostic factor.

### 2.5 Clinical features analysis

To ascertain the clinical relevance of the prognostic risk model generated from the training cohort, we conducted systematic subgroup validation employing the survival and survminer packages in R. This stratified analytical design enabled meticulous evaluation of the model’s predictive accuracy across fundamental clinicopathological parameters, thereby interrogating its robustness and broader applicability within diverse patient demographics. Specifically, stratification analyses were organized according to gender (male vs. female), tumor invasion depth (T1–2 vs. T3–4), nodal status (N0 vs. N1–3), metastatic presence (M0 vs. M1), and overall disease stage (I–II vs. III–IV).

### 2.6 Gene set enrichment analysis

Gene set enrichment analysis (GSEA) was performed to identify differentially activated pathways between high- and low-risk groups in the analysis of the training cohort. Specialized GSEA software was employed to facilitate systematic comparisons of predefined gene sets across these stratified sample groups. To improve interpretability and visual representation of the enrichment results, the ggplot2 package within the R programming environment was utilized for graphical output.

### 2.7 Tumor microenvironment analysis

A thorough characterization of the tumor microenvironment is imperative for deciphering the biological mechanisms that underpin cancer progression and influence therapeutic outcomes. The present study sought to profile the tumor microenvironment in ESCC patients through analysis of transcriptomic data. Stromal, immune, and composite scores were derived for each patient using the limma and estimate packages in R. These scores respectively indicate the abundance of stromal constituents, the degree of immune cell infiltration, and an aggregate estimate of total microenvironmental activity. Differential expression of these scores between high- and low-risk subgroups in the training cohort was statistically evaluated with the limma and ggpubr packages in R.

### 2.8 Immune cell correlation analysis

To characterize the immune landscape of ESCC, multiple deconvolution algorithms—including XCELL, TIMER, EPIC, CIBERSORT-ABS, and CIBERSORT—were employed to investigate associations between immune cell composition, tumor microenvironment features, and riskScores of training cohort. Correlations with a significance level of P < 0.05 were considered statistically significant, with a correlation coefficient > 0 indicating a positive association and < 0 reflecting a negative relationship. All analyses were performed using the following R packages: limma, scales, ggplot2, ggtext, reshape2, tidyverse, and ggpubr.

### 2.9 Single sample gene set enrichment analysis

To comprehensively characterize the immune microenvironment in ESCC, single sample gene set enrichment analysis (ssGSEA) was implemented utilizing the GSVA, limma, and GSEABase packages in R. This analytical framework enabled the quantification of enrichment scores representative of diverse immune cell subpopulations and immune-related functions. Subsequent comparative evaluation of immunological profiles between high- and low-risk patient subgroups within the training cohort was performed with the limma, reshape2, and ggpubr packages, allowing for systematic assessment of differential immune infiltration and functional activity.

### 2.10 Differential analysis of immune checkpoints

Employing R-based computational analytics, a systematic assessment of differential expression profiles among immune checkpoint-associated genes was carried out across risk-stratified subgroups in the training cohort. Utilizing the limma, reshape2, ggplot2, and ggpubr packages, comparative transcriptional profiling was undertaken to identify statistically significant disparities in immune checkpoint gene expression between pre-specified high-risk and low-risk patient subsets.

### 2.11 Single-cell sequencing analysis

The single-cell sequencing data of ESCC (GSE196756) was obtained from the GEO database. This data includes 3 normal samples and 3 tumor samples. Using Seurat (version 5), quality control metrics (nFeature_RNA, nCount_RNA, percent.mt) were calculated. Cells with percent.mt greater than 15% or nFeature_RNA less than 200 were excluded. Standardized expression data was obtained through LogNormalize, and the top 1500 highly variable genes were determined through FindVariableFeatures. Batch correction was performed using harmony (RunHarmony). Graph-based clustering (FindNeighbors and FindClusters) was applied at multiple resolutions, and the stability of the clusters was evaluated using clustree. Cell type annotation for the cells was conducted through SingleR using the celldex reference panel. The annotation results were observed separately in the normal group and the ESCC group.

### 2.12 Drug sensitivity analysis

Employing a systematic computational pharmacogenomic approach with the limma, ggpubr, and pRRophetic packages in R, potential therapeutic agents were stratified based on prognostic risk signatures derived from the training cohort. Application of a stringent statistical threshold (P < 0.05) enabled the identification of compounds exhibiting discriminative sensitivity profiles between high- and low-risk patient subgroups. This risk-informed pharmacogenomic profiling yielded clinically translatable agents that align with individual molecular risk stratifications, offering a framework for personalized treatment strategies in ESCC and supporting refined therapeutic decision-making.

### 2.13 m6A-autophagy-LncRNA regulatory axis

Based on the co-expression network, we first identified m6aRGs and ARGs that exhibited co-expression relationships with the m6aARLncs included in the risk prognosis model. Subsequently, an m6A-autophagy-LncRNA regulatory axis was constructed, integrating these m6aARLncs along with their corresponding m6aRGs and ARGs.

### 2.14 Cell culture

For this study, we used the human EC cell lines KYSE30 and KYSE180 along with normal esophageal epithelial cells (NE2). The two cancer cell lines were cultured in RPMI 1640 medium supplemented with 10% fetal bovine serum (FBS), while the normal epithelial cells were grown in a mixture of Defined Keratinocyte-SFM and Epilife medium. All cells were maintained at 37°C in a humidified incubator with 5% CO_2_ to ensure optimal growth conditions.

### 2.15 Real-time quantitative PCR

Total RNA was isolated from both EC cell lines and normal esophageal epithelial cells (NE2) with TRIzol Reagent (Life Technologies Invitrogen, Catalog No. 15596018) according to the manufacturer’s instructions. The extracted RNA was then reverse-transcribed and amplified using ChamQ Universal SYBR qPCR Master Mix (Vazyme, Cat# Q711-02) following the provided protocol. Real-time quantitative PCR (RT-qPCR) was performed to measure LncRNA expression levels. All primer pairs, which were synthesized by Accurate Biology, are listed in **Table 1**. LncRNA expression was normalized to β-actin as an internal control, and relative expression levels were calculated using the $2^{-\Delta \Delta Ct}$ method.

**Table 1:** Primer sequences for RT-qPCR.

Genes	Forward	Reverse
β-actin	TGGCACCCAGCACAATGAA	CTAAGTCATAGTCCGCCTAGAAGCA
LINC00847	GACTACCACACAAAGACTGCCATC	GGCCTTCCAACTTAGACCAAGC
UBL7-AS1	CCATCCTGTATTCTTCGGACCATG	TTATAGGCCACCACTCTAGGCTCT
LINC01554	AAGCTGCACACGATGACACC	ACGGCCAGTCCTGAATGAGA
LINC00601	TCCACTTGGTGCCCTATGCT	ACCTTCCATGTGTTGCTGCTT
FAM222A-AS1	GGATGCCACCAGATGACACT	CTGTTCCCTGTCATGTCGGT

### 2.16 Statistical analysis

All statistical analyses and accompanying data visualizations were performed using R statistical computing environment (version 4.1.2) and Graphpad Prism software (version 8). Comparisons between two groups were conducted using Student’s t-test, while comparisons involving more than two groups were assessed using one−way analysis of variance (ANOVA). Each experiment was repeated at least three times, and data are presented as mean ± standard deviation. A P−value < 0.05 was considered statistically significant.

## 3 Results

### 3.1 m6a and autophagy-related LncRNAs related to ESCC

**Figure 2:**
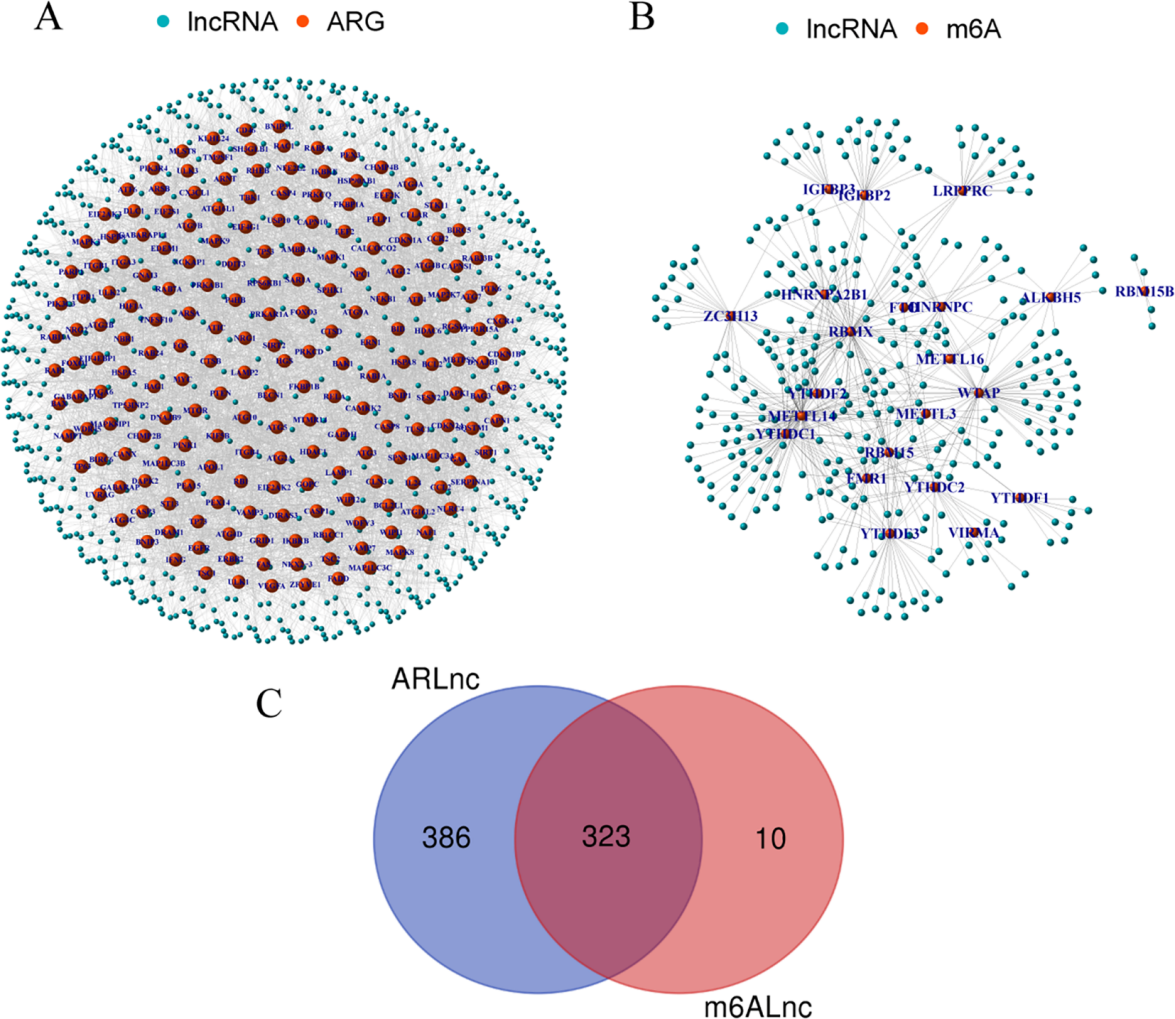
m6aARLncs related to ESCC. **(A)** 709 ARLncs potentially implicated in ESCC pathogenesis. **(B)** 333 m6aRLncs potentially implicated in ESCC pathogenesis. **(C)** 323 m6aARLncs related to ESCC.

Co-expression analysis performed on 222 ARGs and LncRNAs within the ESCC transcriptome identified 709 ARLncs (**Figure 2A**). Similarly, co-expression profiling of 23 m6aRGs and LncRNAs yielded 333 m6aRLncs (**Figure 2B**). Intersection analysis between the 709 ARLncs and 333 m6aRLncs revealed 323 m6aARLncs related with ESCC (**Figure 2C**).

### 3.2 Construction of risk prognostic model

**Figure 3:**
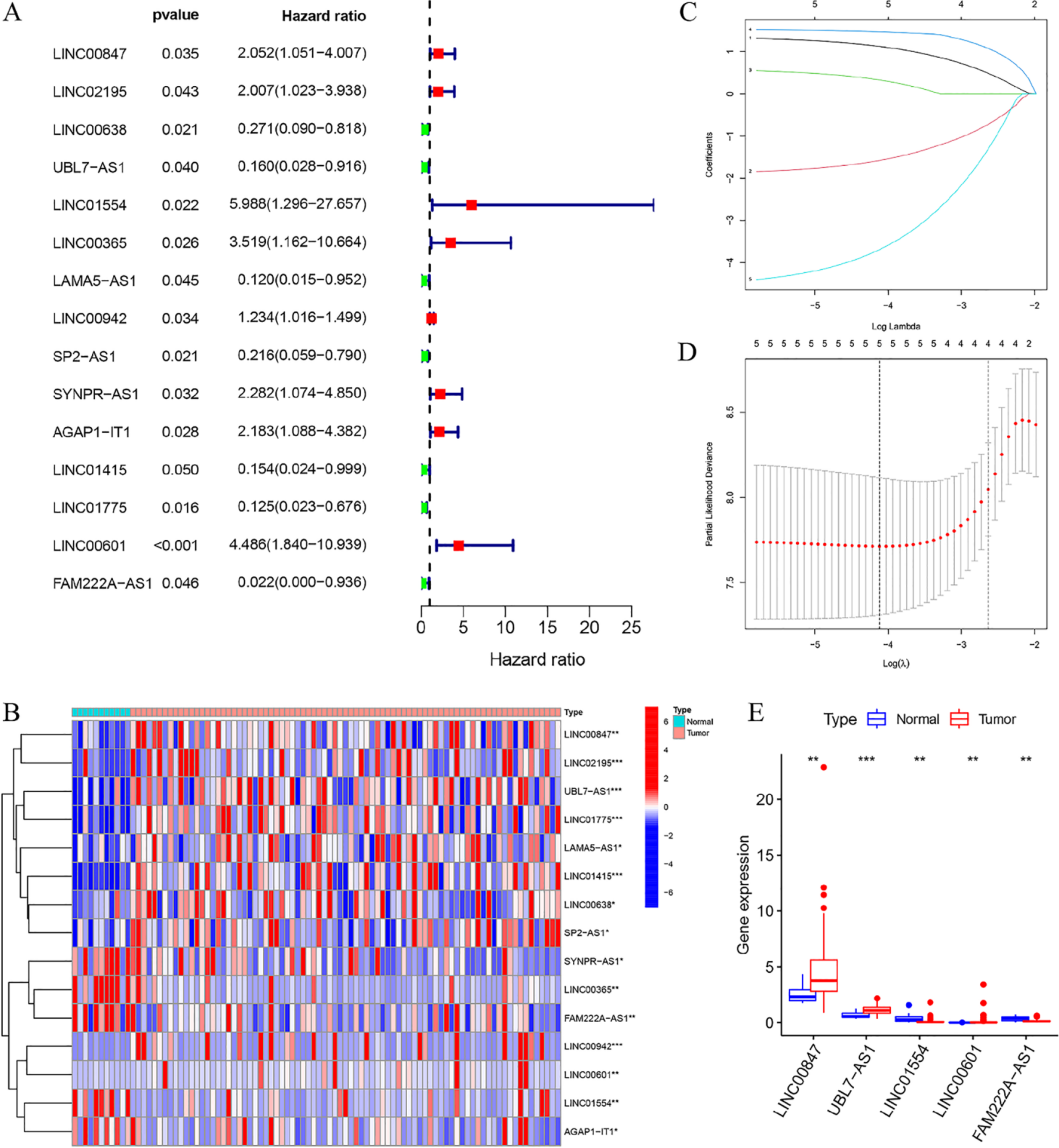
Construction of risk prognostic model. **(A)** Univariate Cox regression analysis obtained 15 candidates prognostic m6aARLncs. **(B)** The difference heatmap of the 15 DE-m6aARLncs. **(C, D)** LASSO regression analysis. **(E)** The difference box plots of the 5 DE-m6aARLncs. *p < 0.05, **p < 0.01, ***p < 0.001.

Through univariate Cox regression analysis of 323 m6aARLncs, we identified 15 m6aARLncs significantly associated with ESCC prognosis (P<0.05) (**Figure 3A**). These m6aARLncs demonstrated differential expression between tumor and adjacent normal tissues and were therefore designated as DE-m6aARLncs (**Figure 3B**). To enhance model generalizability and prevent overfitting, LASSO regression analysis was performed, selecting five optimal prognostic markers (LINC00847, UBL7-AS1, LINC01554, LINC00601, FAM222A-AS1) through minimum cross-validation error criteria (**Figures 3C, D**). Differential expression patterns of these five DE-m6aARLncs were visualized using box plots. LINC00601, UBL7-AS1 and LINC00847 are upregulated in ESCC, while FAM222A-AS1 and LINC01554 are downregulated in ESCC (**Figure 3E**). Using our riskScore algorithm, we computed individual riskScores for all samples and stratified TCGA-ESCC training cohort patients into high-risk (n=40) and low-risk (n=40) groups based on median riskScore. This stratification was successfully validated in an external GEO cohort, which similarly segregated into high-risk (n=60) and low-risk (n=59) groups in GSE53624 (Validation 1), and high-risk (n=90) and low-risk (n=89) groups in GSE53625 (Validation 2).

### 3.3 Validation of the risk prognostic model

**Figure 4:**
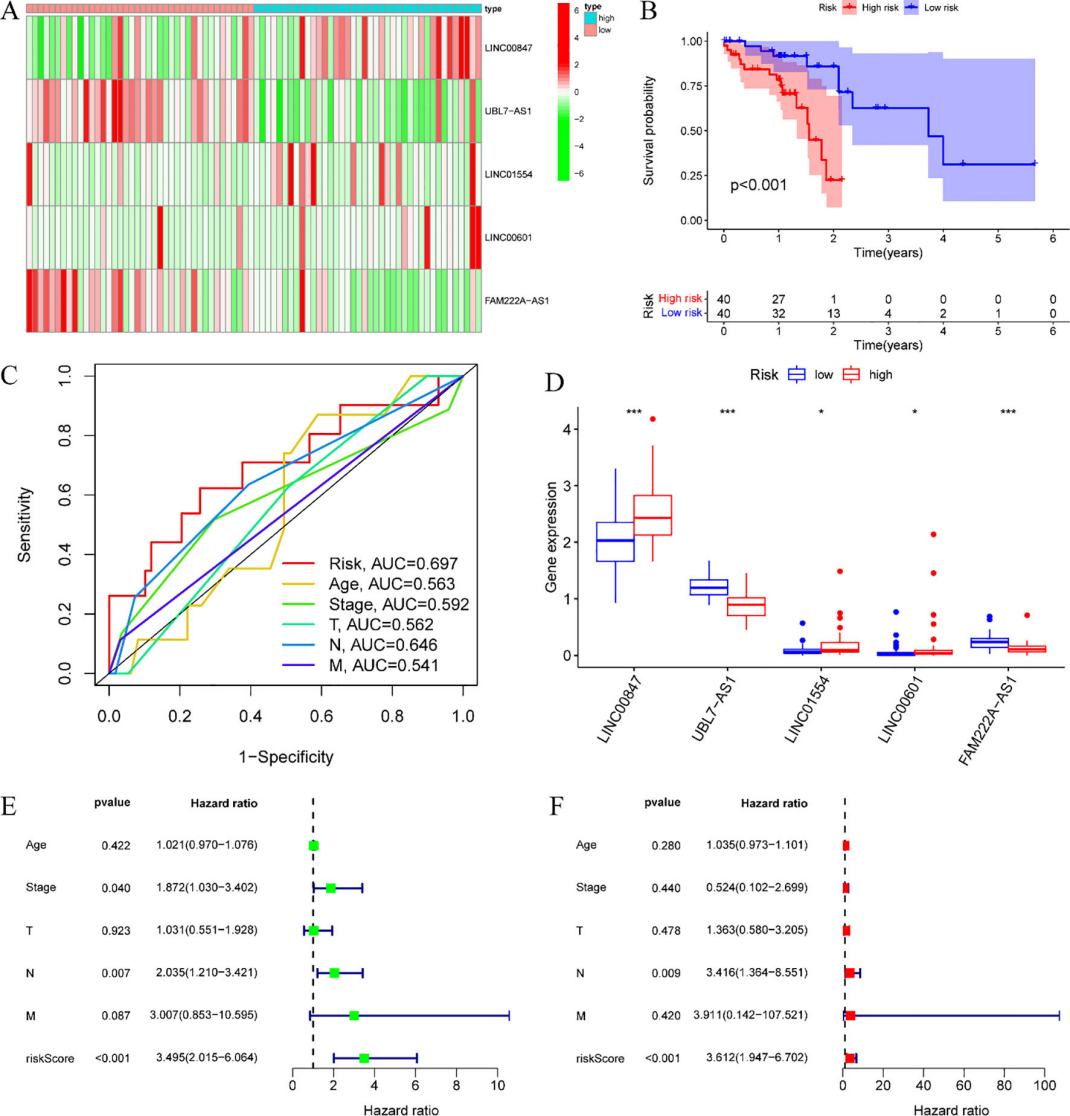
Training cohort. **(A)** Risk heatmap. **(B)** Survival curve. **(C)** ROC curve. **(D)** The differences of 5 DE-m6aARLncs in risk prognostic model. **(E)** Univariate independent prognostic analysis. **(F)** Multivariate independent prognostic analysis. *p < 0.05, ***p < 0.001.

**Figure 5:**
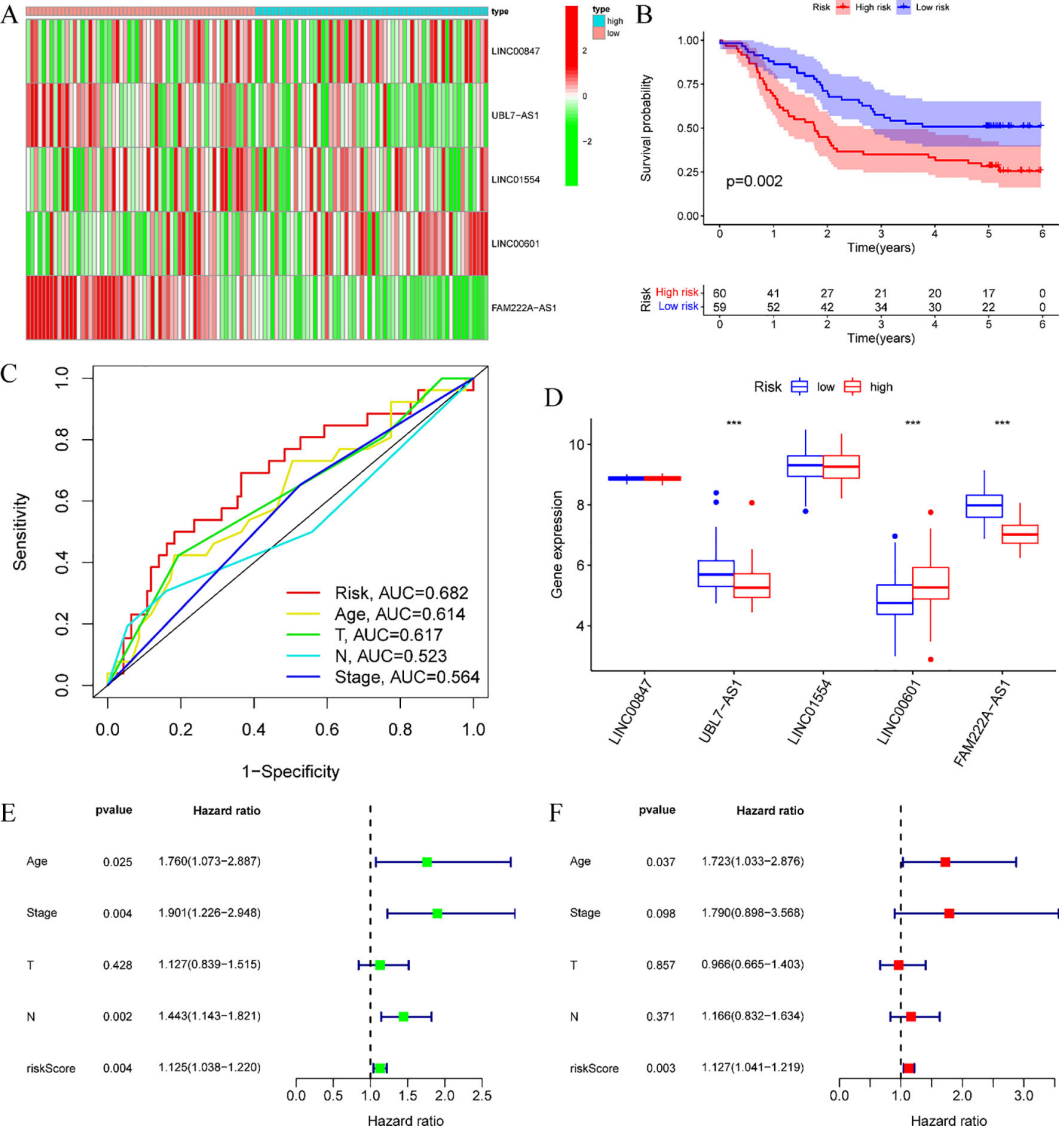
Validation cohort 1. **(A)** Risk heatmap. **(B)** Survival curve. **(C)** ROC curve. **(D)** The differences of 5 DE-m6aARLncs in risk prognostic model. **(E)**Univariate independent prognostic analysis. **(F)** Multivariate independent prognostic analysis. ***p < 0.001.

**Figure 6:**
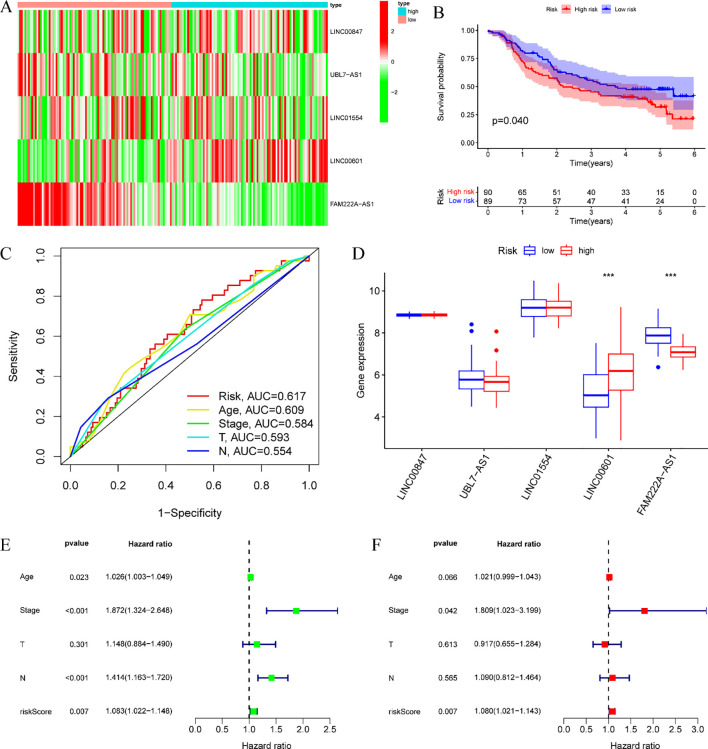
Validation cohort 2. **(A)** Risk heatmap. **(B)** Survival curve. **(C)** ROC curve. **(D)** The differences of 5 DE-m6aARLncs in risk prognostic model. **(E)**Univariate independent prognostic analysis. **(F)** Multivariate independent prognostic analysis. ***p < 0.001.

Expression profiling of the five prognostic DE-m6aARLncs via risk heatmap demonstrated distinct molecular signatures between risk classifications (**Figures 4A**, **5A**, **6A**). Survival analysis confirmed significantly inferior clinical outcomes among high-risk patients across both training and validation cohorts (**Figures 4B**, **5B**, **6B**). ROC curve analysis established the superior predictive performance of the riskScore compared to conventional clinical parameters, confirming its value as an independent prognostic factor. In the training cohort, nodal stage (N stage) emerged as a significant prognostic indicator. In the validation cohort 1, both age and tumor invasion depth (T stage) were identified as clinically relevant prognostic markers; while in the validation cohort 2, age was identified as clinically relevant prognostic markers (**Figures 4C**, **5C**, **6C**). Transcriptional profiling comparing high- and low-risk groups within the training cohort revealed five DE-m6aARLncs—LINC00847, UBL7-AS1, LINC01554, LINC00601, and FAM222A-AS1—exhibiting statistically significant differential expression (**Figure 4D**). This dysregulation pattern was partially conserved in the validation cohort 1, with UBL7-AS1, LINC00601, and FAM222A-AS1 consistently demonstrating risk-correlated expression alterations (**Figure 5D**). LINC00601 and FAM222A-AS1 were validated in the validation cohort 2 (**Figure 6D**). Univariate independent prognostic analysis of the training cohort demonstrated that the riskScore was an independent prognostic factor (p < 0.001) (**Figure 4E**). Multivariate analysis further confirmed its independent prognostic value (p < 0.001) (**Figure 4F**). These findings were consistently validated in both Validation 1 and Validation 2 cohorts, where the riskScore remained an independent prognostic factor for ESCC in both univariate and multivariate analyses (p < 0.01 for each) (**Figures 5E, F**, **6E, F**).

### 3.4 Clinical features analysis

**Figure 7:**
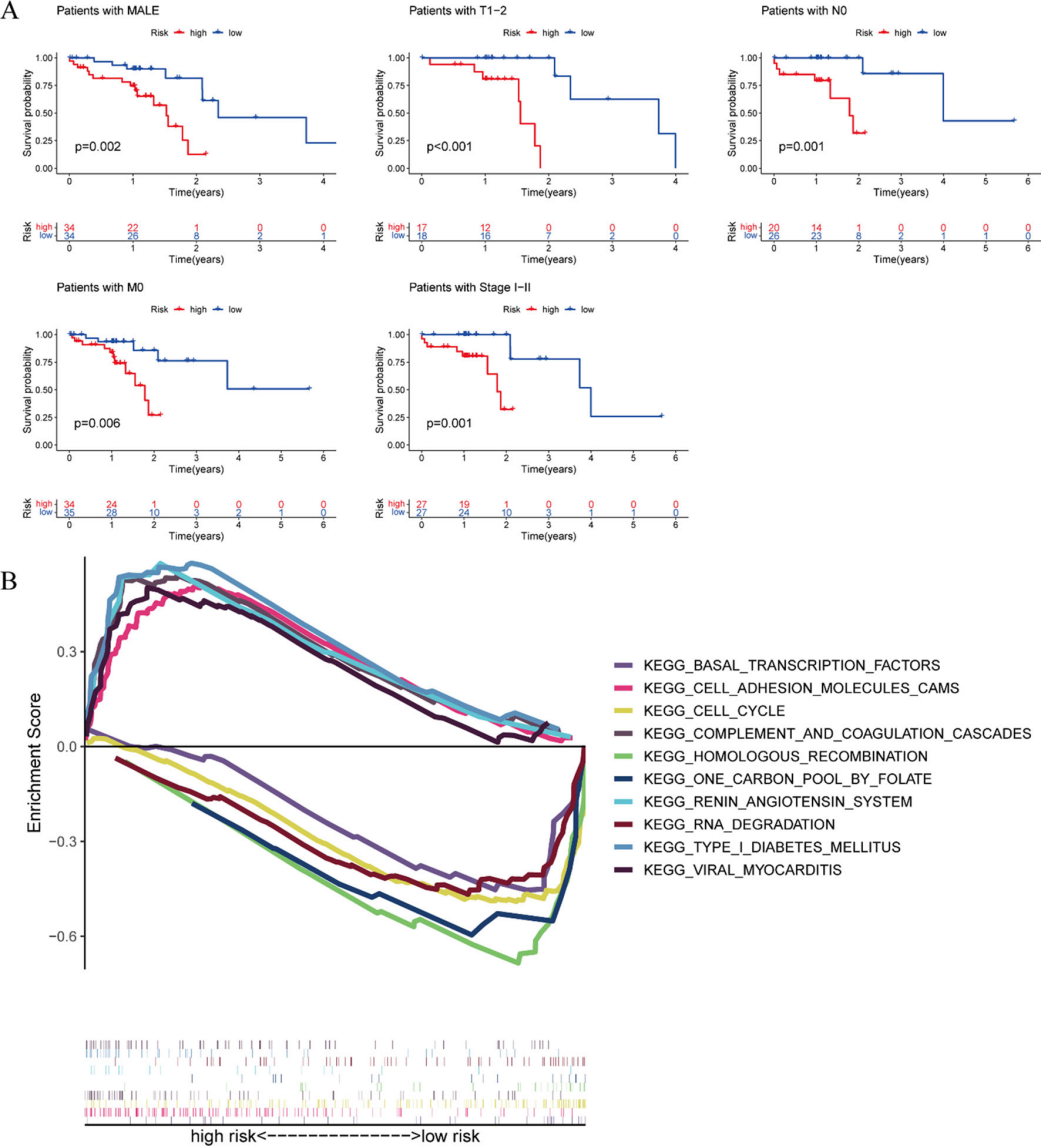
Clinical features analysis and GSEA. **(A)** Clinical features analysis of risk prognostic model. **(B)** GSEA of risk prognostic model.

The risk stratification model consistently demonstrated significant predictive efficacy across clinically relevant subgroups, including male patients and those with T1–2 staging, N0 nodal status, M0 metastasis status, and stage I–II disease (**Figure 7A**). These findings highlight the robustness and broad clinical applicability of the prognostic model within diverse ESCC patient populations.

### 3.5 Gene set enrichment analysis

GSEA revealed distinct pathway activation patterns between risk-stratified subgroups in the training cohort. The high-risk group exhibited significant enrichment in pathways related to cell adhesion molecules cams, renin angiotensin system, type 1 diabetes mellitus, viral myocarditis and complement and coagulation cascades. Conversely, the low-risk group demonstrated prominent activation of pathways associated with homologous recombination, one carbon pool by folate, RNA degradation, cell cycle and basal transcription factors (**Figure 7B**).

### 3.6 Tumor microenvironment analysis

**Figure 8:**
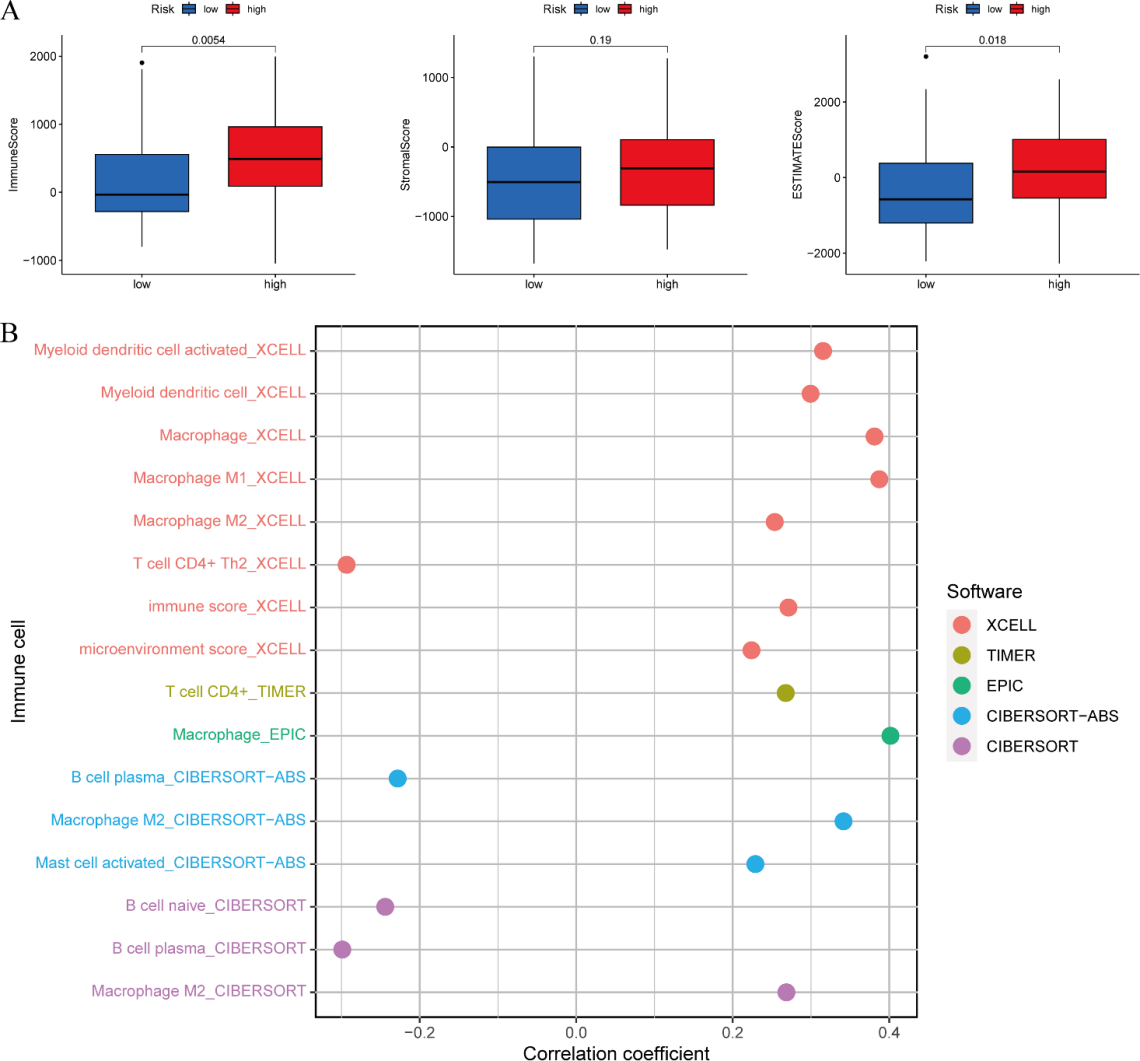
Tumor microenvironment analysis and immune cell correlation analysis. **(A)** Tumor microenvironment analysis of risk prognostic model. **(B)** Immune cell correlation analysis of risk prognostic model.

Analysis of the tumor microenvironment within the prognostic risk model for the training cohort demonstrated statistically significant differences in immune scores and comprehensive microenvironmental scores between high- and low-risk patient subgroups. Notably, both scores were significantly elevated in the high-risk group compared to the low-risk group (**Figure 8A**).

### 3.7 Immune cell correlation analysis

Immune correlation analysis revealed a positive association between riskScore and the infiltration levels of myeloid dendritic cell activated, myeloid dendritic cell, macrophages (including M1 and M2 subtypes), CD4^+^ T cells, and mast cell activated, with elevated abundances of these immune cell types correlating with increased ESCC risk. Conversely, a negative correlation was observed between riskScore and the infiltration of CD4^+^ Th2 cells, B cell plasma and B cell naive, where higher proportions of these cells were associated with reduced ESCC risk. Additionally, riskScore demonstrated positive correlations with both immune and microenvironmental scores (**Figure 8B**).

### 3.8 Single sample gene set enrichment analysis

**Figure 9:**
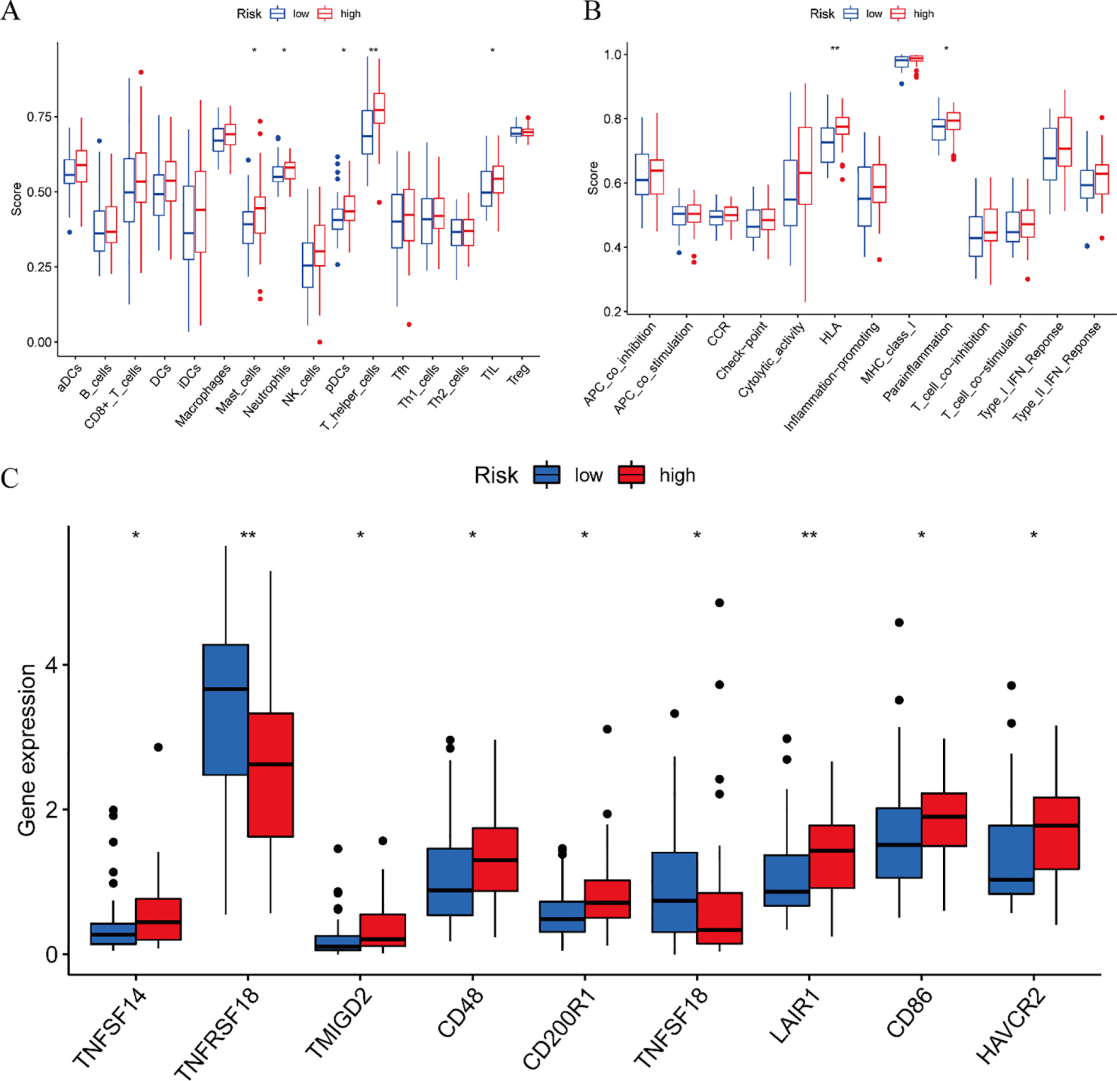
ssGSEA and differential analysis of immune checkpoints. **(A, B)** ssGSEA of risk prognostic model. **(C)** Differential analysis of immune checkpoints for risk prognostic model. *p < 0.05, **p < 0.01.

Comprehensive immune profiling of the risk-stratified cohorts revealed significant enrichment of mast cells, neutrophils, plasmacytoid dendritic cells (pDCs), T helper cells, and tumor-infiltrating lymphocytes (TILs) in high-risk ESCC patients relative to low-risk individuals (**Figure 9A**). Functional characterization further demonstrated elevated activity of human leukocyte antigen (HLA) related pathways and parainflammatory responses in the high-risk subgroup (**Figure 9B**).

### 3.9 Differential analysis of immune checkpoints

A comparative evaluation of immune checkpoint molecule expression identified nine immunoregulatory genes with differential expression patterns between risk-stratified subgroups in the training cohort. The genes TNFSF14, TNFRSF18, TMIGD2, CD48, CD200R1, TNFSF18, LAIR1, CD86, and HAVCR2 demonstrated distinct expression profiles that effectively discriminated high-risk from low-risk ESCC patients, with TNFRSF18 and LAIR1 showing particularly marked differential expression (**Figure 9C**). These findings imply that dysregulation of immune checkpoint mechanisms may be associated with disease advancement and could potentially modulate treatment responsiveness in different prognostic subgroups.

### 3.10 Single-cell sequencing analysis

**Figure 10:**
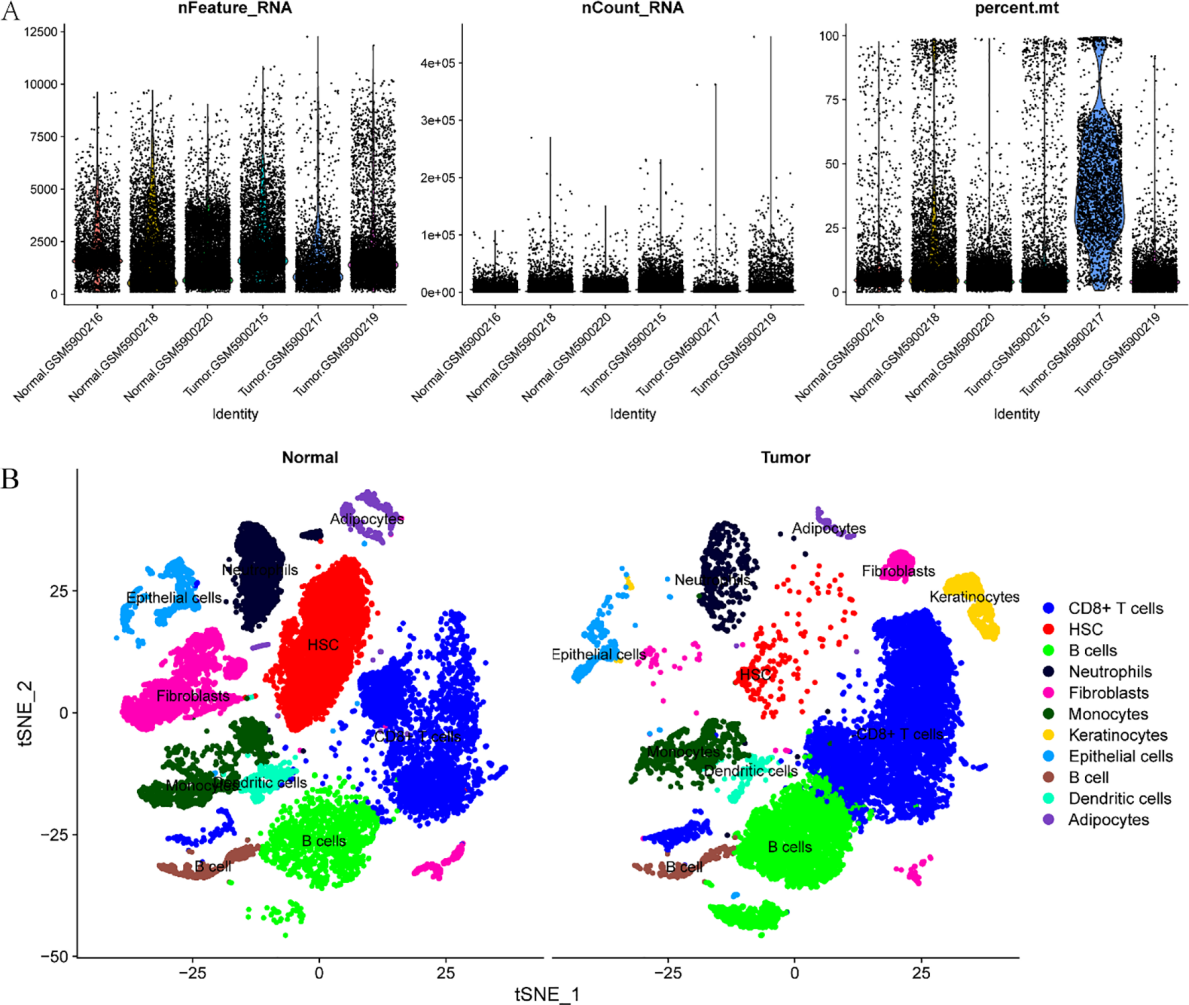
Single-cell sequencing analysis. **(A)** Quality control of single-cell data. **(B)** The results of single-cell sequencing analysis.

Quality control of single-cell data was performed through the three indicators: nFeature_RNA, nCount_RNA and percent.mt (**Figure 10A**). After analyzing and annotating the single-cell sequencing data, 11 types of cells were identified: CD8+ T cells, hematopoietic stem cells (HSC), B cells, neutrophils, fibroblasts, monocytes, keratinocytes, epithelial cells, B cells, dendritic cells, and adipocytes (**Figure 10B**). The B cells, neutrophils and dendritic cells identified in the above immune microenvironment analysis results may have been verified to some extent through single-cell sequencing analysis.

### 3.11 Drug sensitivity analysis

**Figure 11:**
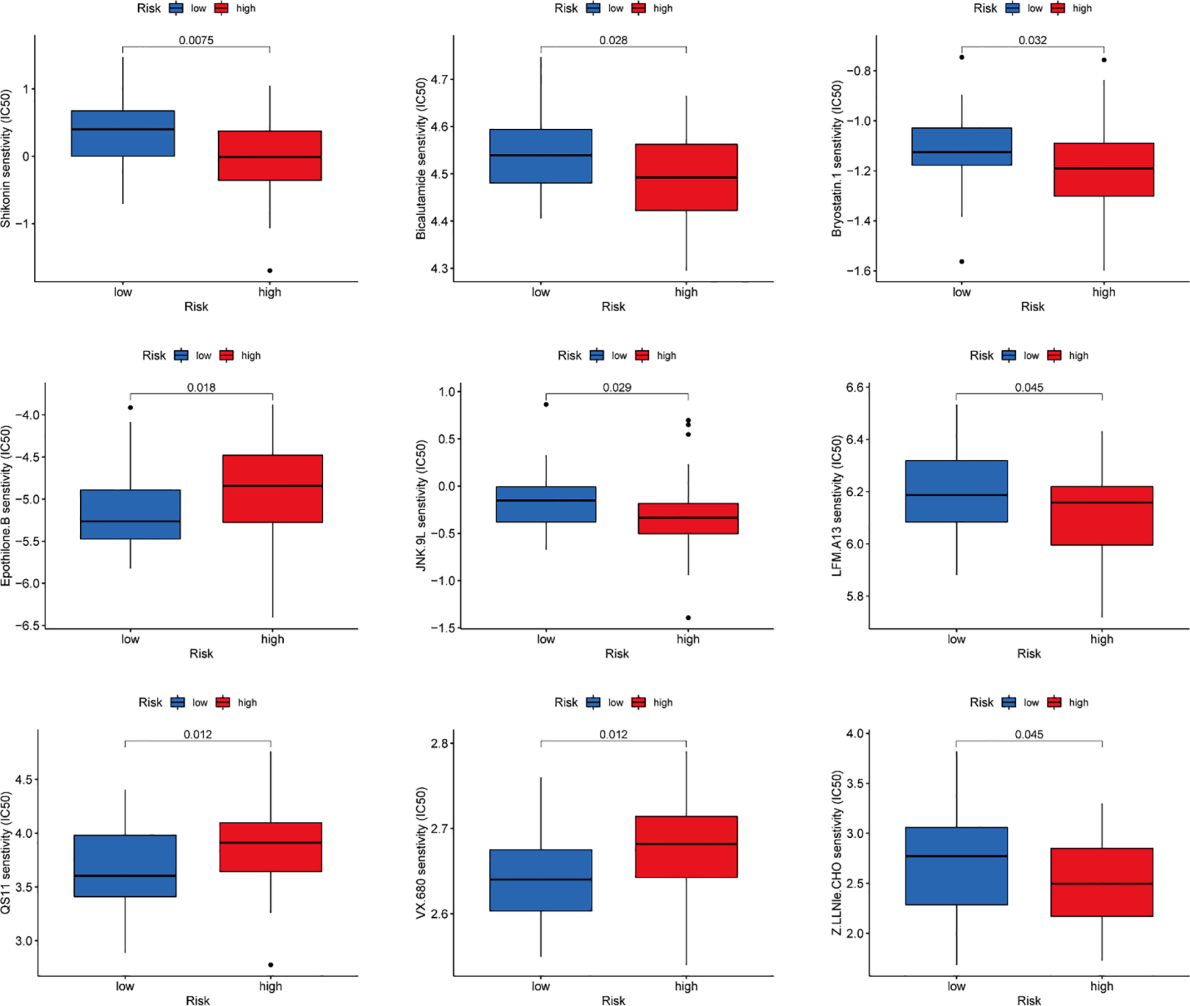
Drug sensitivity analysis.

Through systematic drug sensitivity profiling, nine compounds were identified that exhibited differential efficacy between ESCC prognostic risk groups. Low-risk patients showed increased sensitivity to Shikonin, Bicalutamide, Bryostatin-1, JNK-9L, LFM-A13, and Z-LLNle-CHO, while high-risk patients demonstrated greater responsiveness to Epothilone-B, QS11, and VX-680 (**Figure 11**). These results reveal distinct pharmacogenomic vulnerabilities among risk-stratified ESCC subtypes, providing potential insights for personalized treatment approaches.

### 3.12 m6A-autophagy-LncRNA regulatory axis

**Figure 12:**
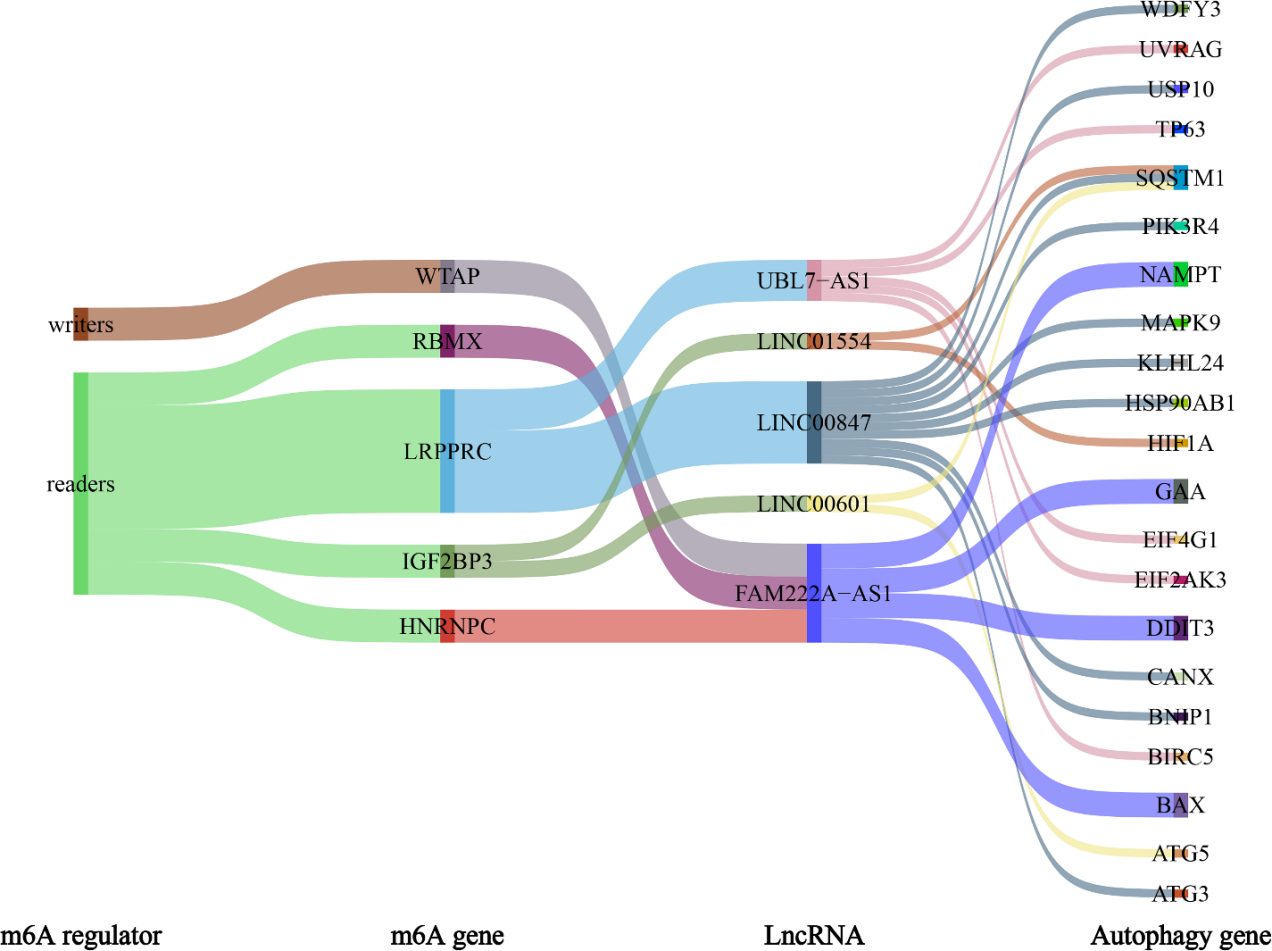
m6A-autophagy-LncRNA regulatory axis.

Comprehensive co-expression analysis identified five m6aRGs and 21 ARGs that exhibited significant co-expression relationships with the five prognostic m6aARLncs incorporated in the risk model. Based on these interactions, we constructed the m6A-autophagy-LncRNA regulatory network to elucidate the potential functional axis among these molecular components (**Figure 12**). These regulatory relationships may play a critical role in the molecular pathogenesis of ESCC.

### 3.13 Validation of the expression of m6aARLncs

**Figure 13:**
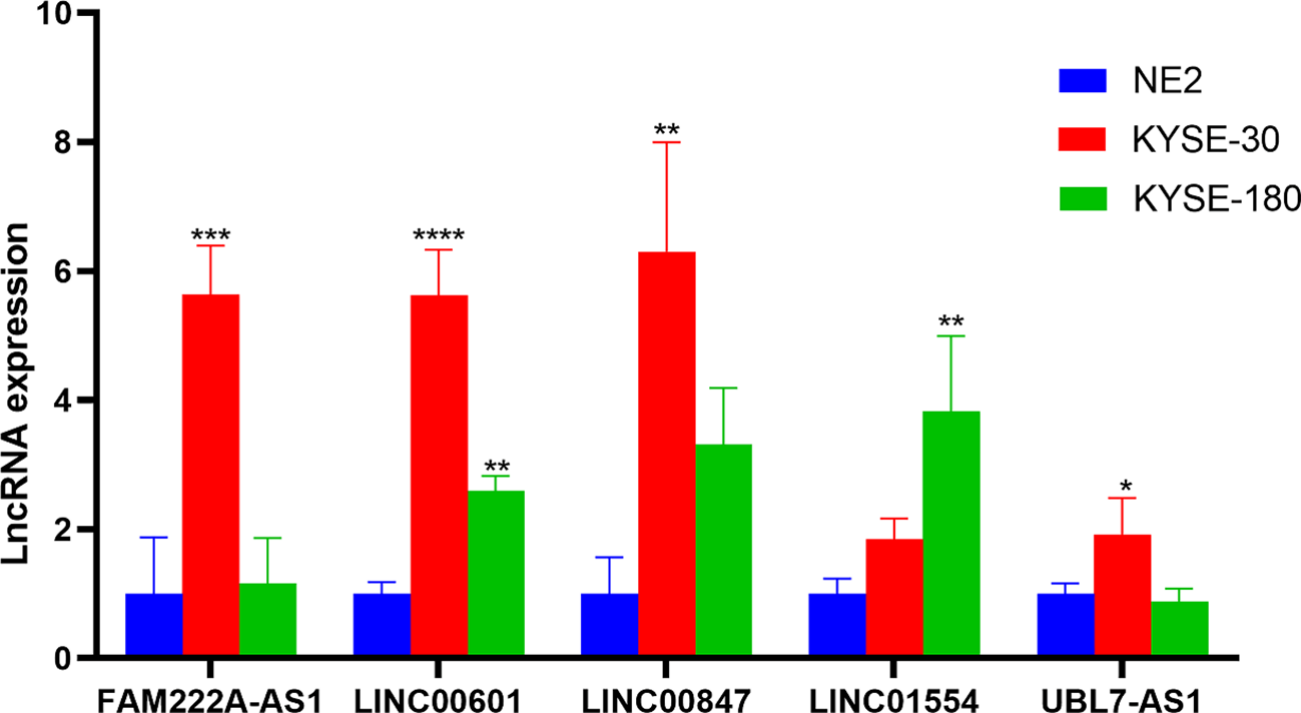
Validation of the mRNA expression level of m6aARLncs. *p< 0.05, **p< 0.01, ***p< 0.001, ****p< 0.0001, each experiment was repeated three times.

To further assess m6aARLncs expression in EC, expression levels were analyzed in two cancer cell lines (KYSE30 and KYSE180) and compared with normal esophageal epithelial cells (NE2) as a control. Relative to NE2 cells, LINC00601 expression was significantly elevated in both KYSE30 and KYSE180. Additionally, FAM222A−AS1, LINC00847, and UBL7−AS1 levels were significantly higher in KYSE30 cells compared to controls, while LINC01554 expression was markedly increased in KYSE180 cells (**Figure 13**).

## 4 Discussion

In this study, we developed a novel prognostic model based on m6aARLncs, which exhibits robust predictive performance for survival outcomes in ESCC patients and offers insights into the regulation of the immune microenvironment. The refined signature consists of five m6aARLncs: LINC00847, UBL7-AS1, LINC01554, LINC00601, and FAM222A-AS1. Comprehensive immune characterization revealed distinct immunophenotypic features in high-risk patients, including elevated infiltration of mast cells, neutrophils, pDCs, T helper cells, and TILs, along with enhanced HLA activity and parainflammatory responses. Additionally, we identified two immune checkpoint molecules—TNFRSF18 and LAIR1—whose expression levels were significantly correlated with risk stratification and may hold translational relevance. Pharmacogenomic evaluation further uncovered nine candidate therapeutic compounds (Shikonin, Bicalutamide, Bryostatin-1, Epothilone-B, JNK-9L, LFM-A13, QS11, VX-680, and Z-LLNle-CHO) demonstrating differential sensitivity between risk subgroups.

LINC00847, located at chromosomal position 5q35.3, has emerged as an oncogenic lncRNA implicated in multiple malignancies through distinct mechanisms regulating proliferation, migration, and invasion. In pancreatic cancer, LINC00847 drives tumor progression via the miR−455−3p/HDAC4 axis (16); in non−small cell lung cancer, E2F1−induced LINC00847 facilitates progression by sponging miR−147a and upregulating IFITM1 (17); and in skin melanoma, it promotes malignant behaviors by targeting the miR−133a−3p/TGFBR1 axis (18). Similarly, the therapy−responsive lncRNAs SNHG1 (resistant) and UBL7−AS1 (sensitive) drive glioblastoma proliferation, with their expression patterns strongly correlating with cell cycle regulators MAD2L1 and CCNB2, respectively (19). In hepatocellular carcinoma, LINC01554 acts as a tumor suppressor; its downregulation has diagnostic and prognostic value, and mechanistic studies demonstrate that its overexpression inhibits viability, migration, and invasion while promoting apoptosis via the miR−148b−3p/EIF4E3 pathway (20, 21). The oncogenic lncRNA LINC00601 promotes glioma progression by activating p−STAT3 signaling, enhancing proliferation and migration (22), whereas FAM222A−AS1 accelerates colorectal cancer progression by antagonizing tumor−suppressive miR−Let−7f, leading to upregulated MYH9 and enhanced metastatic capacity (23). Although these lncRNAs—LINC00847, SNHG1, UBL7−AS1, LINC01554, LINC00601, and FAM222A−AS1—have been characterized in various cancers, their roles in ESCC remain completely unexplored, especially with respect to autophagy and m^6^A modification. The present study demonstrates that these lncRNAs, functioning as m6aARLncs, serve as robust prognostic biomarkers for ESCC patient survival and potentially modulate the tumor immune microenvironment, thereby significantly expanding the current understanding of their biological functions, and opening new avenues for ESCC research and therapeutic development.

TIME has emerged as a critical determinant in the pathogenesis and progression of ESCC, with accumulating evidence highlighting its multifaceted role in tumorigenesis and immune modulation. Recent studies have elucidated that SUMO-modified ETV1 significantly enhances M2−polarized tumor−associated macrophage infiltration and accelerates malignant progression through transcriptional activation of CCL2 in ESCC (24). Furthermore, the Hippo-YAP signaling axis has been implicated in mediating CD24-dependent immune evasion mechanisms by suppressing macrophage phagocytic activity in ESCC (25). Another pivotal finding demonstrates that interleukin-32 (IL-32) encapsulated in extracellular vesicles facilitates M2 macrophage polarization and promotes metastatic dissemination via the FAK/STAT3 signaling cascade (26). Intriguingly, the presence of interleukin-17-expressing mast cells within the muscularis propria has been correlated with improved clinical outcomes, suggesting their potential protective role in ESCC pathogenesis (27). These mast cells exhibit a pro-angiogenic function, as evidenced by their positive correlation with microvessel density, thereby contributing to tumor vascularization in ESCC (28). The prognostic significance of neutrophil infiltration has been established, with elevated intratumoral neutrophil levels and increased peritumoral neutrophil-to-lymphocyte ratio (NLR) serving as independent predictors of adverse outcomes in surgically resected ESCC, underscoring the potential impact of immune dysregulation in disease progression (29). Additionally, the density and distribution of FoxP3+ tumor-infiltrating lymphocytes (TILs) at the invasive margin have been proposed as potential biomarkers for assessing lymph node metastasis risk in superficial ESCC (30). Of particular interest is the identification of HLA-A+ tertiary lymphoid structures (TLSs) within ESCC tissues, where resident TIL-Ts demonstrate upregulated antigen processing machinery (APM) signature expression, indicating their potential for functional reactivation. These TLSs and their cellular constituents, including TIL-Ts and TIL-Bs, may represent novel predictive biomarkers for immune checkpoint blockade therapy response in ESCC patients (31). The tumor depth, CD8+ T cell infiltration, and PD-L1 expression on tumor cells constitute independent prognostic variables in ESCC. The integration of these immune-related parameters into a comprehensive nomogram has significantly enhanced the predictive accuracy for overall survival in post-operative ESCC patients (32). Collectively, these findings emphasize the necessity of a holistic evaluation of TIME components to improve risk stratification and therapeutic decision-making in ESCC management.

The TNFRSF18 gene encodes a critical member of the tumor necrosis factor receptor superfamily (TNFRSF) that demonstrates upregulated expression during T-cell activation. This receptor plays a pivotal role in maintaining immunological self-tolerance through its activity in CD25+CD4+ regulatory T cells (Tregs) (33). Previous genetic studies have characterized regulatory single nucleotide polymorphisms (SNPs) in the TNFRSF18 promoter region among parasite-exposed populations, revealing potential immunomodulatory variations (33). Notably, TNFRSF18 has been implicated in viral pathogenesis through miR-146a-5p-mediated regulation, demonstrating its broader role in immune modulation (34). LAIR1 represents an inhibitory immune checkpoint molecule belonging to both the immunoglobulin superfamily and leukocyte-associated inhibitory receptor family. Expressed ubiquitously on peripheral blood mononuclear cells, LAIR1 serves as a key regulator of immune tolerance through its capacity to suppress T cell function and antigen-presenting cell activity (35). Emerging evidence highlights LAIR1’s tumor-promoting role through multiple mechanisms: its upregulation across various malignancies contributes to immune evasion (24), collagen-mediated LAIR1 activation drives CD8+ T cell exhaustion and confers resistance to PD-1/PD-L1 blockade (36), while in ovarian cancer, LAIR1 suppresses tumor progression via FN1-mediated inhibition of the FAK-MEK-ERK signaling axis (37). Our study makes the novel discovery that both TNFRSF18 and LAIR1 emerge as differentially expressed immune checkpoint genes in ESCC. While current literature lacks report directly linking these molecules to ESCC pathogenesis, our findings establish their significant association with patient prognosis and tumor immune microenvironment modulation. These results provide a compelling foundation for future investigations into TNFRSF18 and LAIR1 as potential therapeutic targets and prognostic biomarkers in ESCC.

Shikonin, a bioactive naphthoquinone derivative isolated from Lithospermum erythrorhizon, exhibits broad-spectrum antitumor activity through multiple mechanisms. In EC, shikonin enhances paclitaxel sensitivity by inducing mitotic arrest and potentiating apoptosis (38), while in EC9706 cells it triggers both autophagy and apoptosis through AMPK/mTOR/ULK1 pathway modulation (39). The anti-androgen Bicalutamide, while primarily employed in prostate cancer therapy (40), demonstrates significant immunomodulatory properties by promoting immunogenic dendritic cell maturation (41) and preserving bone mineral density in osteoporotic patients (42). Bryostatin-1, a potent protein kinase C (PKC) modulator, has reached phase II clinical trials for various malignancies, where it exhibits disease-stabilizing effects (43). Its cytoprotective action in prostate cancer involves regulation of PKC isoform translocation and suppression of PKC-dependent TNF-α release (44). Notably, in advanced EC, sequential administration of Bryostatin.1 with paclitaxel demonstrates clinically meaningful antitumor activity (45). The microtubule-stabilizing agent Epothilone-B induces apoptosis through multiple pathways, including PI3K/AKT/mTOR inhibition (46) and TRAIL/caspase-8 activation in ovarian cancer cells (47), while its semi-synthetic derivative shows radiosensitizing potential in lung cancer (48). Emerging compounds demonstrate diverse antitumor mechanisms: the Bruton’s tyrosine kinase inhibitor LFM-A13 shows enhanced activity against breast cancer when combined with erythropoietin (49, 50); the Wnt/β-catenin pathway activator QS11 exerts dual effects by inhibiting ARFGAP1 protease activity while stimulating Wnt signaling (51); the Aurora kinase inhibitor VX-680 effectively suppresses growth in anaplastic thyroid cancer (52) and metastatic adrenal cortical carcinoma (53); and the proteasome inhibitor Z-LLNle-CHO induces cell death in B-cell acute lymphoblastic leukemia (54). There are currently no clear reports on the related research of JNK-9L. While these compounds have established roles in various malignancies, their therapeutic potential in ESCC remains unexplored. This study provides the first evidence of their possible efficacy against ESCC, representing a significant advancement in the field and warranting further investigation into their clinical applications.

It is important to acknowledge that the proposed m6A−autophagy−LncRNA regulatory network was constructed solely based on co−expression analysis, without incorporating external annotations from specialized databases such as STARBASE, LncBase, or RAID. While co−expression can reveal statistical associations between molecular components, it does not establish directional regulatory relationships or provide evidence for direct physical interactions, such as m6A modification sites on lncRNAs or binding of RNA−binding proteins. Consequently, the biological plausibility of the inferred regulatory axes remains tentative. For example, whether the identified m6A regulatory genes directly modify the predicted lncRNAs, or whether those lncRNAs indeed participate in autophagy−related pathways, has not been substantiated by experimental or database−derived evidence. This limitation may lead to false−positive associations and an overestimation of the regulatory complexity. Future studies should therefore integrate annotation resources that provide experimentally validated or computationally predicted m6A sites, lncRNA−protein interactions, and competing endogenous RNA (ceRNA) networks to refine the regulatory model. Additionally, functional assays such as RNA immunoprecipitation and lncRNA pulldown are necessary to validate the proposed interactions. Until such evidence is obtained, the current network should be viewed as a hypothesis−generating framework rather than a validated mechanistic model.

While this investigation provides novel insights, several limitations warrant consideration. First, the restricted cohort size of ESCC specimens may constrain the statistical power and generalizability of our findings, as the current sample may not fully capture the heterogeneity inherent in ESCC pathogenesis. Future multi-institutional collaborative studies incorporating larger, more diverse patient populations will be essential to validate and refine our predictive model. Second, the proposed m6A−autophagy−LncRNA regulatory network was constructed solely based on co−expression analysis, without incorporating external annotations from specialized databases such as STARBASE, LncBase, or RAID. Third, while we have identified several m6aARLncs with prognostic significance, their precise biological roles in ESCC tumorigenesis remain to be elucidated. Include the molecular mechanisms through which these m6aARLncs mediate post-transcriptional regulation, their immunomodulatory functions within the tumor microenvironment, and their potential interactions with established oncogenic signaling pathways. These will be the important research directions in the future. Fourth, although the report has described the associations between risk characteristics and immune cell populations (such as tumor-infiltrating lymphocytes, plasmacytoid dendritic cells, and mast cells), as well as HLA and inflammatory pathways, the experimental evidence for the results of the immune microenvironment research is limited. Conducting *in vitro* experiments to confirm (such as multiple immunofluorescence, immunohistochemistry, or flow cytometry) as well as confirming these findings at the protein or cellular level are the main contents of our future research. Fifth, nine compounds with different sensitivities in different risk groups have been identified, but they have not been tested in ESCC cell lines or animal models. Future research should conduct functional validation (such as *in vitro* IC50 assays, or xenograft models for certain compounds) to support the clinical translatability of these findings. These limitations, while notable, also present valuable opportunities for advancing the field, and addressing them will significantly enhance our understanding of m6a and autophagy-related mechanisms in ESCC progression and treatment response.

## 5 Conclusion

Through systematic development and validation, this study established a robust prognostic signature comprising five DE-m6aARLncs that demonstrated significant predictive efficacy for ESCC patient outcomes. The model provides mechanistic insights into the interplay between m6A modification, autophagy, and ESCC pathogenesis while enhancing the accuracy of clinical prognosis estimation. Further comprehensive analysis identified distinct immune microenvironment profiles associated with risk stratification, highlighting the potential clinical utility of this signature in informing immunotherapeutic approaches. Pharmacogenomic integration revealed nine therapeutic candidates exhibiting risk-specific sensitivity patterns. These results offer novel directions for targeted therapy development in ESCC. The current research represents a meaningful advancement in precision oncology for ESCC, with relevant implications for prognostic assessment and individualized treatment design. Future clinical validation and translation of these findings may contribute to improved therapeutic strategies and patient outcomes in ESCC care.

## Data Availability

The original contributions presented in the study are included in the article/**Supplementary Material**. Further inquiries can be directed to the corresponding authors.
