# Supplementary material for: Development and validation of an m6A and autophagy related lncRNAs signature for predicting survival and modulating the immune microenvironment in esophageal squamous cell carcinoma
Source: Front Immunol. 2026 May 28;17:1766278. doi: 10.3389/fimmu.2026.1766278 (PMC13254102; doi:10.3389/fimmu.2026.1766278)
Supplement: Supplementary file 1 [file Table1.docx]

library(limma)

expFile="symbol.txt"

geneFile="gene.txt"

setwd("D: \\Exp")

rt=read.table(expFile, header=T, sep="\t", check.names=F)

rt=as.matrix(rt)

rownames(rt)=rt[,1]

exp=rt[,2:ncol(rt)]

dimnames=list(rownames(exp),colnames(exp))

data=matrix(as.numeric(as.matrix(exp)),nrow=nrow(exp),dimnames=dimnames)

data=avereps(data)

data=data[rowMeans(data)>0,]

gene=read.table(geneFile, header=T, check.names=F, sep="\t")

sameGene=intersect(as.vector(gene[,1]), rownames(data))

geneExp=data[sameGene,]

out=rbind(ID=colnames(geneExp),geneExp)

write.table(out,file="m6aGeneExp.txt",sep="\t",quote=F,col.names=F)

library(limma)

corFilter=0.3

pvalueFilter=0.001

setwd("D:\\ARGlncExp")

rt=read.table("lncRNA.txt", header=T, sep="\t", check.names=F)

rt=as.matrix(rt)

rownames(rt)=rt[,1]

exp=rt[,2:ncol(rt)]

dimnames=list(rownames(exp),colnames(exp))

data=matrix(as.numeric(as.matrix(exp)),nrow=nrow(exp),dimnames=dimnames)

data=avereps(data)

data=data[rowMeans(data)>0.1,]

group=sapply(strsplit(colnames(data),"\\-"),"[",4)

group=sapply(strsplit(group,""), "[", 1)

group=gsub("2","1",group)

lncRNA=data[,group==0]

conNum=length(group[group==1])

treatNum=length(group[group==0])

sampleType=c(rep(1,conNum), rep(2,treatNum))

rt1=read.table("NRGexp.txt", header=T, sep="\t", check.names=F)

rt1=as.matrix(rt1)

rownames(rt1)=rt1[,1]

exp1=rt1[,2:ncol(rt1)]

dimnames1=list(rownames(exp1),colnames(exp1))

NRG=matrix(as.numeric(as.matrix(exp1)), nrow=nrow(exp1), dimnames=dimnames1)

NRG=avereps(NRG)

NRG=NRG[rowMeans(NRG)>0.1,]

group=sapply(strsplit(colnames(NRG),"\\-"),"[",4)

group=sapply(strsplit(group,""),"[",1)

group=gsub("2","1",group)

NRG=NRG[,group==0]

outTab=data.frame()

for(i in row.names(lncRNA)){

if(sd(lncRNA[i,])>0.1){

test=wilcox.test(data[i,] ~ sampleType)

if(test$p.value<0.05){

for(j in row.names(NRG)){

x=as.numeric(lncRNA[i,])

y=as.numeric(NRG[j,])

corT=cor.test(x,y)

cor=corT$estimate

pvalue=corT$p.value

if((cor>corFilter) & (pvalue<pvalueFilter)){

outTab=rbind(outTab,cbind(NRG=j,lncRNA=i,cor,pvalue,Regulation="postive"))

}

if((cor< -corFilter) & (pvalue<pvalueFilter)){

outTab=rbind(outTab,cbind(NRG=j,lncRNA=i,cor,pvalue,Regulation="negative"))

}

}

}

}

}

write.table(file="net.network.txt",outTab,sep="\t",quote=F,row.names=F)

lncNode=data.frame(Node=unique(as.vector(outTab[,"lncRNA"])), Type="lncRNA")

mrnaNode=data.frame(Node=unique(as.vector(outTab[,"ARG"])), Type="ARG")

nodeOut=rbind(lncNode, mrnaNode)

write.table(nodeOut, file="net.node.txt", sep="\t", quote=F, row.names=F)

NRGLncRNA=unique(as.vector(outTab[,"lncRNA"]))

NRGLncRNAexp=data[NRGLncRNA,]

NRGLncRNAexp=rbind(ID=colnames(NRGLncRNAexp), NRGLncRNAexp)

write.table(NRGLncRNAexp,file="NRGLncExp.txt",sep="\t",quote=F,col.names=F)

library(igraph)

nodefile="net.node.txt"

edgefile="net.network.txt"

outfile="network.pdf"

lncRNAcol="#00AFBB"

NRGcol="#FC4E07"

setwd("D:\\ network")

node.data=read.table(nodefile, header=T, sep="\t", check.names=F)

edge.data=read.table(edgefile, header=T, sep="\t", check.names=F)

color=ifelse(node.data$Type=="lncRNA", lncRNAcol, NRGcol)

value=ifelse(node.data$Type=="lncRNA", 2, 5)

fontSize=ifelse(node.data$Type=="lncRNA", 0.01, 0.65)

node=data.frame(id=node.data$Node,label=node.data$Node,color=color,shape="dot",value=value,fontSize=fontSize)

edge=data.frame(from=edge.data$lncRNA,to=edge.data$NRG,length=100,arrows="middle",smooth=TRUE,shadow=FALSE,weight=edge.data$cor)

d=data.frame(p1=edge$from, p2=edge$to, weight=abs(edge$weight))

g=graph.data.frame(d,directed = FALSE)

E(g)$color="grey"

V(g)$size=node$value[match(names(components(g)$membership),node$label)]

V(g)$shape="sphere"

V(g)$lable.cex=node$fontSize[match(names(components(g)$membership),node$label)]

V(g)$color=node$color[match(names(components(g)$membership),node$label)]

pdf(outfile, width=8, height=7)

layout(mat=matrix(c(1,2,1,2),nc=2), height=c(1,11))

par(mar=c(0,0,0,0))

plot(1,type="n",axes=F,xlab="",ylab="")

legend('center',legend=c('lncRNA','ARG'),col=c(lncRNAcol,NRGcol),pch=16,bty="n",ncol=2,cex=2)

vertex.frame.color = node$color

edge_col=E(g)$color

plot(g,layout=layout_on_sphere,vertex.size=V(g)$size,vertex.label=node$label,vertex.label.cex=V(g)$lable.cex,edge.width =0.05,edge.arrow.size=0,vertex.label.color=NULL,vertex.frame.color=NA,edge.color=edge_col,vertex.label.font=2)

dev.off()

library(limma)

expFile="symbol.txt"

geneFile="gene.txt"

setwd("D:\\ m6aExp")

rt=read.table(expFile, header=T, sep="\t", check.names=F)

rt=as.matrix(rt)

rownames(rt)=rt[,1]

exp=rt[,2:ncol(rt)]

dimnames=list(rownames(exp),colnames(exp))

data=matrix(as.numeric(as.matrix(exp)),nrow=nrow(exp),dimnames=dimnames)

data=avereps(data)

data=data[rowMeans(data)>0,]

gene=read.table(geneFile, header=T, check.names=F, sep="\t")

sameGene=intersect(as.vector(gene[,1]), rownames(data))

geneExp=data[sameGene,]

out=rbind(ID=colnames(geneExp),geneExp)

write.table(out,file="m6aGeneExp.txt",sep="\t",quote=F,col.names=F)

library(survival)

pFilter=0.05

setwd("D:\\uniCox")

rt=read.table("expTime.txt",header=T,sep="\t",check.names=F,row.names=1)

outTab=data.frame()

sigGenes=c("futime","fustat")

rt[,3:ncol(rt)]=log2(rt[,3:ncol(rt)]+1)

for(i in colnames(rt[,3:ncol(rt)])){

cox <- coxph(Surv(futime, fustat) ~ rt[,i], data = rt)

coxSummary = summary(cox)

coxP=coxSummary$coefficients[,"Pr(>|z|)"]

if(coxP<pFilter){

sigGenes=c(sigGenes,i)

outTab=rbind(outTab,

cbind(id=i,

HR=coxSummary$conf.int[,"exp(coef)"],

HR.95L=coxSummary$conf.int[,"lower .95"],

HR.95H=coxSummary$conf.int[,"upper .95"],

pvalue=coxSummary$coefficients[,"Pr(>|z|)"])

)

}

}

write.table(outTab,file="uniCox.txt",sep="\t",row.names=F,quote=F)

uniSigExp=rt[,sigGenes]

uniSigExp=cbind(id=row.names(uniSigExp),uniSigExp)

write.table(uniSigExp,file="uniSigExp.txt",sep="\t",row.names=F,quote=F)

rt <- read.table("uniCox.txt",header=T,sep="\t",row.names=1,check.names=F)

gene <- rownames(rt)

hr <- sprintf("%.3f",rt$"HR")

hrLow <- sprintf("%.3f",rt$"HR.95L")

hrHigh <- sprintf("%.3f",rt$"HR.95H")

Hazard.ratio <- paste0(hr,"(",hrLow,"-",hrHigh,")")

pVal <- ifelse(rt$pvalue<0.001, "<0.001", sprintf("%.3f", rt$pvalue))

pdf(file="forest.pdf", width = 6.5,height =7)

n <- nrow(rt)

nRow <- n+1

ylim <- c(1,nRow)

layout(matrix(c(1,2),nc=2),width=c(3,2))

xlim = c(0,3)

par(mar=c(4,2.5,2,1))

plot(1,xlim=xlim,ylim=ylim,type="n",axes=F,xlab="",ylab="")

text.cex=0.8

text(0,n:1,gene,adj=0,cex=text.cex)

text(1.5-0.5*0.2,n:1,pVal,adj=1,cex=text.cex);text(1.5-0.5*0.2,n+1,'pvalue',cex=text.cex,font=2,adj=1)

text(3,n:1,Hazard.ratio,adj=1,cex=text.cex);text(3,n+1,'Hazard ratio',cex=text.cex,font=2,adj=1,)

par(mar=c(4,1,2,1),mgp=c(2,0.5,0))

xlim = c(0,max(as.numeric(hrLow),as.numeric(hrHigh)))

plot(1,xlim=xlim,ylim=ylim,type="n",axes=F,ylab="",xaxs="i",xlab="Hazard ratio")

arrows(as.numeric(hrLow),n:1,as.numeric(hrHigh),n:1,angle=90,code=3,length=0.05,col="darkblue",lwd=2.5)

abline(v=1,col="black",lty=2,lwd=2)

boxcolor = ifelse(as.numeric(hr) > 1, 'red', 'green')

points(as.numeric(hr), n:1, pch = 15, col = boxcolor, cex=1.3)

axis(1)

dev.off()

library("glmnet")

library("survival")

coxSigFile="tcga.uniSigExp.txt"

geoFile="geo.expTime.txt"

rt=read.table(coxSigFile, header=T, sep="\t", check.names=F, row.names=1)

geo=read.table(geoFile, header=T, sep="\t", check.names=F, row.names=1)

sameGene=intersect(colnames(rt)[3:ncol(rt)], colnames(geo)[3:ncol(geo)])

rt=rt[,c("futime","fustat",sameGene)]

rt$futime[rt$futime<=0]=0.003

x=as.matrix(rt[,c(3:ncol(rt))])

y=data.matrix(Surv(rt$futime, rt$fustat))

fit=glmnet(x, y, family="cox", maxit=1000)

pdf("lasso.lambda.pdf")

plot(fit, xvar = "lambda", label = TRUE)

dev.off()

cvfit=cv.glmnet(x, y, family="cox", maxit=1000)

pdf("lasso.cvfit.pdf")

plot(cvfit)

abline(v=log(c(cvfit$lambda.min,cvfit$lambda.1se)),lty="dashed")

dev.off()

coef=coef(fit, s=cvfit$lambda.min)

index=which(coef != 0)

actCoef=coef[index]

lassoGene=row.names(coef)[index]

geneCoef=cbind(Gene=lassoGene, Coef=actCoef)

write.table(geneCoef, file="lasso.geneCoef.txt", sep="\t", quote=F, row.names=F)

trainFinalGeneExp=rt[,lassoGene]

myFun=function(x){crossprod(as.numeric(x),actCoef)}

trainScore=apply(trainFinalGeneExp,1,myFun)

outCol=c("futime","fustat",lassoGene)

risk=as.vector(ifelse(trainScore>median(trainScore),"high","low"))

outTab=cbind(rt[,outCol],riskScore=as.vector(trainScore),risk)

write.table(cbind(id=rownames(outTab),outTab),file="trainRisk.txt",sep="\t",quote=F,row.names=F)

rt=read.table(geoFile, header=T, sep="\t", check.names=F, row.names=1)

rt$futime=rt$futime/365

testFinalGeneExp=rt[,lassoGene]

testScore=apply(testFinalGeneExp,1,myFun)

outCol=c("futime","fustat",lassoGene)

risk=as.vector(ifelse(testScore>median(trainScore),"high","low"))

outTab=cbind(rt[,outCol],riskScore=as.vector(testScore),risk)

write.table(cbind(id=rownames(outTab),outTab),file="testRisk.txt",sep="\t",quote=F,row.names=F)

library(survival)

library(survminer)

setwd("D:\\ survival")

bioSurvival=function(inputFile=null,outFile=null){

rt=read.table(inputFile, header=T, sep="\t", check.names=F)

diff=survdiff(Surv(futime, fustat) ~risk,data = rt)

pValue=1-pchisq(diff$chisq,df=1)

if(pValue<0.001){

pValue="p<0.001"

}else{

pValue=paste0("p=",sprintf("%.03f",pValue))

}

fit <- survfit(Surv(futime, fustat) ~ risk, data = rt)

surPlot=ggsurvplot(fit,

data=rt,

conf.int=T,

pval=pValue,

pval.size=6,

legend.title="Risk",

legend.labs=c("High risk", "Low risk"),

xlab="Time(years)",

break.time.by = 1,

palette=c("red", "blue"),

risk.table=TRUE,

risk.table.title="",

risk.table.height=.25)

pdf(file=outFile,onefile = FALSE,width = 6.5,height =5.5)

print(surPlot)

dev.off()

}

bioSurvival(inputFile="trainRisk.txt", outFile="trainSurv.pdf")

bioSurvival(inputFile="testRisk.txt", outFile="testSurv.pdf")

library(pheatmap)

setwd("D:\\ riskPlot")

bioRiskPlot=function(inputFile=null,riskScoreFile=null,survStatFile=null,heatmapFile=null){

rt=read.table(inputFile, header=T, sep="\t", check.names=F, row.names=1)

rt=rt[order(rt$riskScore),]

riskClass=rt[,"risk"]

lowLength=length(riskClass[riskClass=="low"])

highLength=length(riskClass[riskClass=="high"])

lowMax=max(rt$riskScore[riskClass=="low"])

line=rt[,"riskScore"]

line[line>10]=10

pdf(file=riskScoreFile, width=7, height=4)

plot(line, type="p", pch=20,

xlab="Patients (increasing risk socre)", ylab="Risk score",

col=c(rep("green",lowLength),rep("red",highLength)) )

abline(h=lowMax,v=lowLength,lty=2)

legend("topleft", c("High risk", "Low Risk"),bty="n",pch=19,col=c("red","green"),cex=1.2)

dev.off()

color=as.vector(rt$fustat)

color[color==1]="red"

color[color==0]="green"

pdf(file=survStatFile, width=7, height=4)

plot(rt$futime, pch=19,

xlab="Patients (increasing risk socre)", ylab="Survival time (years)",

col=color)

legend("topleft", c("Dead", "Alive"),bty="n",pch=19,col=c("red","green"),cex=1.2)

abline(v=lowLength,lty=2)

dev.off()

rt1=rt[c(3:(ncol(rt)-2))]

rt1=t(rt1)

annotation=data.frame(type=rt[,ncol(rt)])

rownames(annotation)=rownames(rt)

pdf(file=heatmapFile, width=7, height=4)

pheatmap(rt1,

annotation=annotation,

cluster_cols = FALSE,

cluster_rows = FALSE,

show_colnames = F,

scale="row",

color = colorRampPalette(c(rep("green",3.5), "white", rep("red",3.5)))(50),

fontsize_col=3,

fontsize=7,

fontsize_row=8)

dev.off()

}

bioRiskPlot(inputFile="trainRisk.txt",riskScoreFile="train.riskScore.pdf",survStatFile="train.survStat.pdf",heatmapFile="train.heatmap.pdf")

bioRiskPlot(inputFile="testRisk.txt",riskScoreFile="test.riskScore.pdf",survStatFile="test.survStat.pdf",heatmapFile="test.heatmap.pdf")

library(survival)

library(survminer)

library(timeROC)

setwd("D:\\ ROC")

bioROC=function(inputFile=null, rocFile=null){

predictTime=1

rt=read.table(inputFile, header=T, sep="\t")

ROC_rt=timeROC(T=rt$futime, delta=rt$fustat,

marker=rt$riskScore, cause=1,

weighting='aalen',

times=c(predictTime), ROC=TRUE)

pdf(file=rocFile, width=5, height=5)

plot(ROC_rt, time=predictTime, col='red', title=FALSE, lwd=2)

legend('bottomright', cex=1.3,

paste0('AUC=',sprintf("%.03f",ROC_rt$AUC[2])),

col="white", lwd=1, bty = 'n')

dev.off()

}

bioROC(inputFile="trainRisk.txt",rocFile="train.ROC.pdf")

bioROC(inputFile="testRisk.txt",rocFile="test.ROC.pdf")

library(survival)

library(survminer)

riskFile="allRisk.txt"

cliFile="clinical.txt"

setwd("D:\\ cliGroupSur")

risk=read.table(riskFile, header=T, sep="\t", check.names=F, row.names=1)

cli=read.table(cliFile, header=T, sep="\t", check.names=F, row.names=1)

sameSample=intersect(row.names(cli), row.names(risk))

risk=risk[sameSample,]

cli=cli[sameSample,]

data=cbind(futime=risk[,1],fustat=risk[,2],cli,risk=risk[,"risk"])

for(i in colnames(data[,3:(ncol(data)-1)])){

rt=data[,c("futime","fustat",i,"risk")]

rt=rt[(rt[,i]!="unknow"),]

colnames(rt)=c("futime","fustat","clinical","risk")

tab=table(rt[,"clinical"])

tab=tab[tab!=0]

for(j in names(tab)){

rt1=rt[(rt[,"clinical"]==j),]

tab1=table(rt1[,"risk"])

tab1=tab1[tab1!=0]

labels=names(tab1)

if(length(labels)==2){

titleName=j

if((i=="age") | (i=="Age") | (i=="AGE")){

titleName=paste0("age",j)

}

diff=survdiff(Surv(futime, fustat) ~risk,data = rt1)

pValue=1-pchisq(diff$chisq,df=1)

if(pValue<0.001){

pValue="p<0.001"

}else{

pValue=paste0("p=",sprintf("%.03f",pValue))

}

fit <- survfit(Surv(futime, fustat) ~ risk, data = rt1)

#????????????

surPlot=ggsurvplot(fit,

data=rt1,

conf.int=F,

pval=pValue,

pval.size=6,

title=paste0("Patients with ",titleName),

legend.title="Risk",

legend.labs=labels,

font.legend=12,

xlab="Time(years)",

break.time.by = 1,

palette=c("red", "blue"),

risk.table=TRUE,

risk.table.title="",

risk.table.col = "strata",

risk.table.height=.25)

j=gsub(">=","ge",j);j=gsub("<=","le",j);j=gsub(">","gt",j);j=gsub("<","lt",j)

pdf(file=paste0("survival.",i,"_",j,".pdf"),onefile = FALSE,

width = 6,

height =5)

print(surPlot)

dev.off()

}

}

}

install.packages("ggpubr")

library(limma)

library(ggpubr)

scoreFile="TMEscores.txt"

riskFile="risk.all.txt"

setwd("D:\\TMEdiff")

rt=read.table(scoreFile, header=T, sep="\t", check.names=F, row.names=1)

data=as.matrix(rt)

rownames(data)=gsub("(.*?)\\-(.*?)\\-(.*?)\\-(.*?)\\-.*", "\\1\\-\\2\\-\\3", rownames(data))

data=avereps(data)

risk=read.table(riskFile, header=T, sep="\t", check.names=F, row.names=1)

sameSample=intersect(row.names(data), row.names(risk))

data=data[sameSample,,drop=F]

risk=risk[sameSample,"risk",drop=F]

rt=cbind(data, risk)

rt$risk=factor(rt$risk, levels=c("low", "high"))

group=levels(factor(rt$risk))

rt$risk=factor(rt$risk, levels=group)

comp=combn(group,2)

my_comparisons=list()

for(i in 1:ncol(comp)){my_comparisons[[i]]<-comp[,i]}

for(i in colnames(rt)[1:3]){

boxplot=ggboxplot(rt, x="risk", y=i, fill="risk",

xlab="",

ylab=i,

legend.title="Risk",

palette=c("#0066FF","#FF0000")

)+

stat_compare_means(comparisons=my_comparisons)

pdf(file=paste0(i, ".pdf"), width=5, height=4.5)

print(boxplot)

dev.off()

}

library(limma)

library(scales)

library(ggplot2)

library(ggtext)

library(reshape2)

library(tidyverse)

library(ggpubr)

riskFile="risk.all.txt"

immFile="infiltration_estimation_for_tcga.csv"

setwd("C:\\immuneCor")

risk=read.table(riskFile, header=T, sep="\t", check.names=F, row.names=1)

immune=read.csv(immFile, header=T, sep=",", check.names=F, row.names=1)

immune=as.matrix(immune)

rownames(immune)=gsub("(.*?)\\-(.*?)\\-(.*?)\\-(.*)", "\\1\\-\\2\\-\\3", rownames(immune))

immune=avereps(immune)

sameSample=intersect(row.names(risk), row.names(immune))

risk=risk[sameSample, "riskScore"]

immune=immune[sameSample,]

x=as.numeric(risk)

x[x>quantile(x,0.99)]=quantile(x,0.99)

outTab=data.frame()

for(i in colnames(immune)){

y=as.numeric(immune[,i])

if(sd(y)<0.001){next}

corT=cor.test(x, y, method="spearman")

cor=corT$estimate

pvalue=corT$p.value

if(pvalue<0.05){

outTab=rbind(outTab,cbind(immune=i, cor, pvalue))

outFile=paste0("cor.", i, ".pdf")

outFile=gsub("/", "_", outFile)

df1=as.data.frame(cbind(x,y))

p1=ggplot(df1, aes(x, y)) +

xlab("Risk score") + ylab(i)+

geom_point() + geom_smooth(method="lm",formula = y ~ x) + theme_bw()+

stat_cor(method = 'spearman', aes(x =x, y =y))

pdf(file=outFile, width=5, height=4.7)

print(p1)

dev.off()

}

}

write.table(file="corResult.txt", outTab, sep="\t", quote=F, row.names=F)

corResult=read.table("corResult.txt", head=T, sep="\t")

corResult$Software=sapply(strsplit(corResult[,1],"_"), '[', 2)

corResult$Software=factor(corResult$Software,level=as.character(unique(corResult$Software[rev(order(as.character(corResult$Software)))])))

b=corResult[order(corResult$Software),]

b$immune=factor(b$immune,levels=rev(as.character(b$immune)))

colslabels=rep(hue_pal()(length(levels(b$Software))),table(b$Software))

pdf(file="correlation.pdf", width=9, height=6)

ggplot(data=b, aes(x=cor, y=immune, color=Software))+

labs(x="Correlation coefficient",y="Immune cell")+

geom_point(size=4.1)+

theme(panel.background=element_rect(fill="white",size=1,color="black"),

panel.grid=element_line(color="grey75",size=0.5),

axis.ticks = element_line(size=0.5),

axis.text.y = ggtext::element_markdown(colour=rev(colslabels)))

dev.off()

library(limma)

library(reshape2)

library(ggpubr)

setwd("D:\\ scoreCor")

scoreCor=function(riskFile=null, scoreFile=null, project=null){

data=read.table(scoreFile, header=T, sep="\t", check.names=F, row.names=1)

data=t(data)

risk=read.table(riskFile, header=T, sep="\t", check.names=F, row.names=1)

sameSample=intersect(row.names(data),row.names(risk))

data=data[sameSample,,drop=F]

risk=risk[sameSample,,drop=F]

rt=cbind(data,risk[,c("riskScore","risk")])

rt=rt[,-(ncol(rt)-1)]

immCell=c("aDCs","B_cells","CD8+_T_cells","DCs","iDCs","Macrophages",

"Mast_cells","Neutrophils","NK_cells","pDCs","T_helper_cells",

"Tfh","Th1_cells","Th2_cells","TIL","Treg")

rt1=rt[,c(immCell,"risk")]

data=melt(rt1,id.vars=c("risk"))

colnames(data)=c("Risk","Type","Score")

data$Risk=factor(data$Risk, levels=c("low","high"))

p=ggboxplot(data, x="Type", y="Score", color = "Risk",

xlab="",ylab="Score",add = "none",palette = c("blue","red") )

p=p+rotate_x_text(50)

p=p+stat_compare_means(aes(group=Risk),symnum.args=list(cutpoints = c(0, 0.001, 0.01, 0.05, 1), symbols = c("***", "**", "*", "")),label = "p.signif")

#????ͼƬ?ļ?

pdf(file=paste0(project,".immCell.pdf"), width=7, height=6)

print(p)

dev.off()

immFunction=c("APC_co_inhibition","APC_co_stimulation","CCR",

"Check-point","Cytolytic_activity","HLA","Inflammation-promoting",

"MHC_class_I","Parainflammation","T_cell_co-inhibition",

"T_cell_co-stimulation","Type_I_IFN_Reponse","Type_II_IFN_Reponse")

rt1=rt[,c(immFunction,"risk")]

data=melt(rt1,id.vars=c("risk"))

colnames(data)=c("Risk","Type","Score")

data$Risk=factor(data$Risk, levels=c("low","high"))

p=ggboxplot(data, x="Type", y="Score", color = "Risk",

xlab="",ylab="Score",add = "none",palette = c("blue","red") )

p=p+rotate_x_text(50)

p=p+stat_compare_means(aes(group=Risk),symnum.args=list(cutpoints = c(0, 0.001, 0.01, 0.05, 1), symbols = c("***", "**", "*", "")),label = "p.signif")

#????ͼƬ?ļ?

pdf(file=paste0(project,".immFunction.pdf"), width=7, height=6)

print(p)

dev.off()

}

scoreCor(riskFile="trainRisk.txt", scoreFile="TCGA.score.txt", project="TCGA")

scoreCor(riskFile="testRisk.txt", scoreFile="GEO.score.txt", project="GEO")

library(limma)

library(reshape2)

library(ggplot2)

library(ggpubr)

expFile="symbol.txt"

riskFile="risk.txt"

geneFile="gene.txt"

setwd("D:\\0000 ")

rt=read.table(expFile, header=T, sep="\t", check.names=F)

rt=as.matrix(rt)

rownames(rt)=rt[,1]

exp=rt[,2:ncol(rt)]

dimnames=list(rownames(exp),colnames(exp))

data=matrix(as.numeric(as.matrix(exp)),nrow=nrow(exp),dimnames=dimnames)

data=avereps(data)

gene=read.table(geneFile, header=F, sep="\t", check.names=F)

sameGene=intersect(row.names(data),as.vector(gene[,1]))

data=t(data[sameGene,])

data=log2(data+1)

group=sapply(strsplit(row.names(data),"\\-"),"[",4)

group=sapply(strsplit(group,""),"[",1)

group=gsub("2","1",group)

data=data[group==0,]

row.names(data)=gsub("(.*?)\\-(.*?)\\-(.*?)\\-(.*?)\\-.*","\\1\\-\\2\\-\\3",row.names(data))

data=avereps(data)

risk=read.table(riskFile, sep="\t", header=T, check.names=F, row.names=1)

sameSample=intersect(row.names(data),row.names(risk))

rt1=cbind(data[sameSample,],risk[sameSample,])

rt1=rt1[,c(sameGene,"risk")]

sigGene=c()

for(i in colnames(rt1)[1:(ncol(rt1)-1)]){

if(sd(rt1[,i])<0.001){next}

wilcoxTest=wilcox.test(rt1[,i] ~ rt1[,"risk"])

pvalue=wilcoxTest$p.value

if(wilcoxTest$p.value<0.05){

sigGene=c(sigGene, i)

}

}

sigGene=c(sigGene, "risk")

rt1=rt1[,sigGene]

rt1=melt(rt1,id.vars=c("risk"))

colnames(rt1)=c("risk","Gene","Expression")

group=levels(factor(rt1$risk))

rt1$risk=factor(rt1$risk, levels=c("low","high"))

comp=combn(group,2)

my_comparisons=list()

for(j in 1:ncol(comp)){my_comparisons[[j]]<-comp[,j]}

boxplot=ggboxplot(rt1, x="Gene", y="Expression", fill="risk",

xlab="",

ylab="Gene expression",

legend.title="Risk",

width=0.8,

palette = c("#0066FF", "#FF0000") )+

rotate_x_text(50)+

stat_compare_means(aes(group=risk),

method="wilcox.test",

symnum.args=list(cutpoints=c(0, 0.001, 0.01, 0.05, 1), symbols=c("***", "**", "*", "ns")), label="p.signif")

pdf(file="checkpoint.diff.pdf", width=8, height=5)

print(boxplot)

dev.off()

install.packages("ggpubr")

library(limma)

library(ggpubr)

library(pRRophetic)

library(ggplot2)

set.seed(12345)

pFilter=0.05

expFile="symbol.txt"

riskFile="risk.all.txt"

setwd("D:\\ pRRophetic")

allDrugs=c("A.443654", "A.770041", "ABT.263", "ABT.888", "AG.014699", "AICAR", "AKT.inhibitor.VIII", "AMG.706", "AP.24534", "AS601245", "ATRA", "AUY922", "Axitinib", "AZ628", "AZD.0530", "AZD.2281", "AZD6244", "AZD6482", "AZD7762", "AZD8055", "BAY.61.3606", "Bexarotene", "BI.2536", "BIBW2992", "Bicalutamide", "BI.D1870", "BIRB.0796", "Bleomycin", "BMS.509744", "BMS.536924", "BMS.708163", "BMS.754807", "Bortezomib", "Bosutinib", "Bryostatin.1", "BX.795", "Camptothecin", "CCT007093", "CCT018159", "CEP.701", "CGP.082996", "CGP.60474", "CHIR.99021", "CI.1040", "Cisplatin", "CMK", "Cyclopamine", "Cytarabine", "Dasatinib", "DMOG", "Docetaxel", "Doxorubicin", "EHT.1864", "Elesclomol", "Embelin", "Epothilone.B", "Erlotinib", "Etoposide", "FH535", "FTI.277", "GDC.0449", "GDC0941", "Gefitinib", "Gemcitabine", "GNF.2", "GSK269962A", "GSK.650394", "GW.441756", "GW843682X", "Imatinib", "IPA.3", "JNJ.26854165", "JNK.9L", "JNK.Inhibitor.VIII", "JW.7.52.1", "KIN001.135", "KU.55933", "Lapatinib", "Lenalidomide", "LFM.A13", "Metformin", "Methotrexate", "MG.132", "Midostaurin", "Mitomycin.C", "MK.2206", "MS.275", "Nilotinib", "NSC.87877", "NU.7441", "Nutlin.3a", "NVP.BEZ235", "NVP.TAE684", "Obatoclax.Mesylate", "OSI.906", "PAC.1", "Paclitaxel", "Parthenolide", "Pazopanib", "PD.0325901", "PD.0332991", "PD.173074", "PF.02341066", "PF.4708671", "PF.562271", "PHA.665752", "PLX4720", "Pyrimethamine", "QS11", "Rapamycin", "RDEA119", "RO.3306", "Roscovitine", "Salubrinal", "SB.216763", "SB590885", "Shikonin", "SL.0101.1", "Sorafenib", "S.Trityl.L.cysteine", "Sunitinib", "Temsirolimus", "Thapsigargin", "Tipifarnib", "TW.37", "Vinblastine", "Vinorelbine", "Vorinostat", "VX.680", "VX.702", "WH.4.023", "WO2009093972", "WZ.1.84", "X17.AAG", "X681640", "XMD8.85", "Z.LLNle.CHO", "ZM.447439")

rt = read.table(expFile, header=T, sep="\t", check.names=F)

rt=as.matrix(rt)

rownames(rt)=rt[,1]

exp=rt[,2:ncol(rt)]

dimnames=list(rownames(exp),colnames(exp))

data=matrix(as.numeric(as.matrix(exp)),nrow=nrow(exp),dimnames=dimnames)

data=avereps(data)

data=data[rowMeans(data)>0.5,]

group=sapply(strsplit(colnames(data),"\\-"), "[", 4)

group=sapply(strsplit(group,""), "[", 1)

group=gsub("2","1",group)

data=data[,group==0]

data=t(data)

rownames(data)=gsub("(.*?)\\-(.*?)\\-(.*?)\\-(.*)", "\\1\\-\\2\\-\\3", rownames(data))

data=avereps(data)

data=t(data)

riskRT=read.table(riskFile, header=T, sep="\t", check.names=F, row.names=1)

for(drug in allDrugs){

senstivity=pRRopheticPredict(data, drug, selection=1)

senstivity=senstivity[senstivity!="NaN"]

#senstivity[senstivity>quantile(senstivity,0.99)]=quantile(senstivity,0.99)

sameSample=intersect(row.names(riskRT), names(senstivity))

risk=riskRT[sameSample, "risk",drop=F]

senstivity=senstivity[sameSample]

rt=cbind(risk, senstivity)

rt$risk=factor(rt$risk, levels=c("low", "high"))

type=levels(factor(rt[,"risk"]))

comp=combn(type, 2)

my_comparisons=list()

for(i in 1:ncol(comp)){my_comparisons[[i]]<-comp[,i]}

test=wilcox.test(senstivity~risk, data=rt)

if(test$p.value<pFilter){

boxplot=ggboxplot(rt, x="risk", y="senstivity", fill="risk",

xlab="Risk",

ylab=paste0(drug, " senstivity (IC50)"),

legend.title="Risk",

palette=c("#0066FF","#FF0000")

)+

stat_compare_means(comparisons=my_comparisons)

pdf(file=paste0("durgSenstivity.", drug, ".pdf"), width=5, height=4.5)

print(boxplot)

dev.off()

}

}
